# Metabolic and Evolutionary Insights in the Transformation of Diphenylamine by a *Pseudomonas putida* Strain Unravelled by Genomic, Proteomic, and Transcription Analysis

**DOI:** 10.3389/fmicb.2018.00676

**Published:** 2018-04-06

**Authors:** Evangelia S. Papadopoulou, Chiara Perruchon, Sotirios Vasileiadis, Constantina Rousidou, Georgia Tanou, Martina Samiotaki, Athanassios Molassiotis, Dimitrios G. Karpouzas

**Affiliations:** ^1^Department of Biochemistry and Biotechnology, Laboratory of Plant and Environmental Biotechnology, University of Thessaly, Larissa, Greece; ^2^School of Agriculture, Aristotle University of Thessaloniki, Thessaloniki, Greece; ^3^Biomedical Sciences Research Center “Alexander Fleming”, Vari, Greece

**Keywords:** diphenylamine, *Pseudomonas putida*, biodegradation, metabolic pathway, genomic and proteomic analysis

## Abstract

Diphenylamine (DPA) is a common soil and water contaminant. A *Pseudomonas putida* strain, recently isolated from a wastewater disposal site, was efficient in degrading DPA. Thorough knowledge of the metabolic capacity, genetic stability and physiology of bacteria during biodegradation of pollutants is essential for their future industrial exploitation. We employed genomic, proteomic, transcription analyses and plasmid curing to (i) identify the genetic network of *P. putida* driving the microbial transformation of DPA and explore its evolution and origin and (ii) investigate the physiological response of bacterial cells during degradation of DPA. Genomic analysis identified (i) two operons encoding a biphenyl (bph) and an aniline (tdn) dioxygenase, both flanked by transposases and (ii) two operons and several scattered genes encoding the *ortho*-cleavage of catechol. Proteomics identified 11 putative catabolic proteins, all but BphA1 up-regulated in DPA- and aniline-growing cells, and showed that the bacterium mobilized cellular mechanisms to cope with oxidative stress, probably induced by DPA and its derivatives. Transcription analysis verified the role of the selected genes/operons in the metabolic pathway: DPA was initially transformed to aniline and catechol by a biphenyl dioxygenase (DPA-dioxygenase); aniline was then transformed to catechol which was further metabolized via the *ortho*-cleavage pathway. Plasmid curing of *P. putida* resulted in loss of the DPA and aniline dioxygenase genes and the corresponding degradation capacities. Overall our findings provide novel insights into the evolution of the DPA degradation pathway and suggests that the degradation capacity of *P. putida* was acquired through recruitment of the *bph* and *tdn* operons via horizontal gene transfer.

## Introduction

Diphenylamine (DPA) is an industrial chemical used as a precursor in the production of azo-dyes (Lye and Freeman, 1999) or non-steroidal anti-inflammatory drugs (Masubuchi et al., 1999) and also, due to its strong antioxidant properties, as stabilizer for propellants (Drzyzga, 2003) and as preservative in fruit-packaging plants (Rudell et al., 2005). The latter two applications have been deemed responsible for the long-term contamination of soil, surface water and groundwater systems with DPA (Entenmann and Schacke, 1994; Tsaboula et al., 2016). Considering the high aquatic toxicity of DPA (Drzyzga, 2003; EFSA, 2012), its widespread occurrence in water resources constitute a serious environmental problem. Remediation of contaminated sites and treatment of DPA-contaminated effluents are still top environmental priorities. The use of microorganisms for the bioremediation and biodepuration of DPA contaminated sites and effluents could be a worth-pursuing strategy.

DPA as a secondary amine is generally considered non-biodegradable (EFSA, 2012). Hence little information is available regarding its microbial degradation. Shin and Spain (2009) provided the first and only to date report on the isolation of a DPA-degrading bacterium, a *Burkholderia* sp. strain JS667. The operons involved in the degradation of DPA resided in a composite transposon, however its localization in the bacterial genome and the function of the genes and enzymes encoded in this transposon were not determined. Perruchon et al. (2015) recently described the isolation of a DPA-degrading *Pseudomonas putida* strain DPA1, which was able to utilize DPA as a carbon and nitrogen source and degrade it via the intermediate formation of aniline and catechol. However the full metabolic pathway and the enzymes involved are still not known. Good knowledge of the (i) intermediate and final transformation products derived from DPA biodegradation, (ii) phenotypic stability and (iii) homeostasis of *P. putida* cells during the biodegradation process are essential for its future industrial exploitation.

Recent advances in sequencing technologies enabled the genomic analysis of bacterial strains with interesting catabolic properties. This facilitated the prediction of pollutants transformation pathways (Trivedi et al., 2016; Yan et al., 2016), which could be further validated via proteomic (Vandera et al., 2015) or transcriptomic analysis (Gunasekera et al., 2017). Combination of omics approaches enables the detailed study of the catabolic mechanisms driving the degradation of organic pollutants and their regulatory network in the context of the overall cellular response of the bacterial strain during the biodegradation processes (Vilchez-Vargas et al., 2010; Bers et al., 2011), an aspect which has remained unexplored in the biodegradation of DPA.

The aims of the present work were (a) to explore the genetic network driving the transformation of DPA by *P. putida* DPA1, in the frame of its overall cellular response to DPA exposure, and (b) to determine the localization and organization of the catabolic operons in the genome of *P. putida* DPA1, shedding light into the evolution of the transformation pathway. To achieve these goals genomic, proteomic, and transcription analyses complemented by plasmid curing were employed. The genome of *P. putida* was assembled, annotated and operons with a putative role in the transformation of DPA were identified. Proteomic analysis determined differentially expressed proteins (in the presence of DPA, aniline or succinate) associated either with the transformation of DPA or the homeostasis of bacterial cells. The role of putative catabolic enzymes in the transformation of DPA was confirmed by reverse transcription (RT)-q-PCR. The localization of the key catabolic operons in the bacterial genome was further explored via plasmid curing.

## Materials and methods

### Bacterial strain, growth conditions, chemicals and analytical methods

A *P. putida* strain DPA1 able to rapidly degrade DPA (Perruchon et al., 2015) was used in the present study. The bacterium was routinely grown at 26°C/180 rpm on a mineral salt medium supplemented with nitrogen (MSMN) and amended with filter sterilized DMSO solutions of DPA (50 g L^−1^) to a final concentration of 25 mg L^−1^. Aniline, succinate and catechol solutions in DMSO (1–10 g L^−1^) were used for amending the growth medium with these chemicals. The DMSO concentration in all media did not exceed 0.3%. Analytical standards of DPA (99.9% purity) and catechol (≥99%) were purchased from Sigma-Aldrich (St Louis, USA), aniline (≥99%) from ChemLab (Phoenix, USA) and succinate (99%) from PanReac-AppliChem (St. Louis, USA). Bacterial growth was determined by optical density at 600 nm (OD_600_). The degradation of DPA, aniline and catechol in MSMN was determined by HPLC-UV (Perruchon et al., 2015).

### Genomic analysis of *P. putida* DPA1

Total bacterial DNA was extracted from an actively growing *P. putida* DPA1 culture with the Purelink Genomic DNA Mini kit (Invitrogen Life Technologies, USA) and quantified by Qubit (Fisher Scientific, USA). Sequencing was performed by Illumina MiSeq with a 2 × 300 bp paired-end (insert ~550 bp) and a 2 × 300 bp mate-pair (insert ~3000 bp) runs. Genome assembly was performed with Allpaths-LG v50960 (Ribeiro et al., 2012) using the default parameters. Genome completeness and purity was checked with the CheckM v0.9.6 software suite (Parks et al., 2015) and annotation of the resulting contigs was performed with Prokka v1.10 (Seemann, 2014) using various programs as described in detail by Perruchon et al. (2017). The genome annotation was further curated according to best BLAST hits against the NCBI nt and RefSeq databases in the case that homologs were missing from the default Prokka databases. The assembled genome of the strain DPA1 has been deposited at NCBI under the accession number NNBI00000000 (BioProject #: PRJNA386862). Gene organization visualization was performed with the genoPoltR v0.8.6 (Guy et al., 2010) package of the R v3.3.2 software (R Core Team, 2015).

### Phylogenetic analyses of catabolic enzymes

Predicted proteins from the genome of *P. putida* strain DPA1 with a putative role in the transformation of DPA were subjected to maximum likelihood phylogenetic analysis. Briefly, closely related protein sequences were retrieved from NCBI using the basic local alignment search tool (BLAST) software v2.2.27+ (Altschul et al., 1990) while querying our sequence of interest against the non-redundant (nr) NCBI database. The retrieved sequences together with the corresponding query were aligned with Muscle v3.7 (Edgar, 2004) and informative alignment blocks were retained for further analysis using GBlocks v0.91b (Talavera and Castresana, 2007). ProtTest v3.4 (Abascal et al., 2005) was used for selecting the best evolutionary model according to the Akaike information criterion values (AIC), and a maximum likelihood tree was generated according to that model using the RAxML software v8.1.24 (Stamatakis, 2014) with 1000 bootstrap replicates as described previously (Perruchon et al., 2017).

### Proteomic analysis of *P. putida* strain DPA1

#### Experimental set up and crude protein extraction

*Pseudomonas putida* DPA1 was inoculated at an OD_600_ of 0.02 in triplicate cultures of MSMN amended with DPA (100 mg L^−1^), aniline (110 mg L^−1^) and succinate (212 mg L^−1^), aiming to an equivalent concentration of carbon (85 mg C L^−1^) in the three treatments. Duplicate non-inoculated DPA and aniline controls were also prepared to check the abiotic degradation of these compounds. All samples were incubated as described above. The degradation of DPA and aniline and bacterial growth was followed in all cultures by HPLC-UV and OD_600_measurement respectively. When bacterial growth in all treatments reached at the mid-log phase, bacterial cells were harvested by centrifugation (5,000 rpm, 10 min, 4°C) and re-suspended in cold buffer A (50 mM tris-base, 100 mM NaCl, 10% glycerol, pH 7.5). Cells were mixed with 0.1% TRITON and 0.5 mM PMSF and ultrasonicated on ice three times for 15 s. Cell debris were removed by centrifugation (12,000 rpm, 45 min, 4°C) and further processed for proteomic analysis as described below.

#### 2-D proteomic analysis and protein identification by mass spectrometry

For 2D-PAGE separation crude protein extracts were further clarified, concentrated and protein pellet was dissolved in rehydration buffer as described by Ziogas et al. (2015). Protein concentration was determined using a Bio-Rad assay kit with BSA as standard (Bradford, 1976). Protein extracts were then analyzed by 2D-PAGE as described by Ainalidou et al. (2016). For each replicate sample (three biological replicates per treatment were run in parallel) 30 μg of total soluble proteins were analyzed. Proteins were initially separated by isoelectric focusing using gel strips forming an immobilized non-linear pH gradient from 3 to 10 (pH 3–10 NL IPG strips, 11 cm; Bio-Rad) and then by SDS–PAGE using 12.5% Tris-HCl polyacrylamide gels (Bio-Rad) following standard procedures. 2-D gels were silver stained and scanned with Bio-Rad GS-800 Calibrated Densitometer equipped with PDQuest Advanced 2-D Gel Analysis software (version 8.1, Bio-Rad) as described by Ziogas et al. (2015). Data were analyzed by one-way ANOVA (*P* ≤ 0.05) and means were compared using Student's *t*-test (significance level 95%). The statistical significant differences were further combined by the quantitative 2-fold change of spot volume. Spots showing values in the ratios DPA/Succinate and aniline/Succinate (volume intensity) lower than 0.5 or higher than 2 were excised from the 2D-PAGE gels and digested with 0.01 μg μL^−1^ trypsin (Roche Diagnostics, Switzerland) for 16 h at room temperature. Tryptic peptide mixtures (1 μL) were analyzed in a MALDI-TOF mass spectrometer (Autoflex-Speed, Bruker Daltonics). The protein identification was carried out by peptide mass fingerprinting on a locally installed Mascot-Server v 2.0 against the genome of *P. putida* DPA1 & Uniprot-Trembl databases. Proteins not identified by MALDI-TOF analysis were reanalyzed by HPLC-tandem MS/MS (Thermo Scientific) as described previously (Perruchon et al., 2017).

### Transcriptional analysis of putative catabolic genes

In the proteomic experiment described above, bacterial pellet collected at 1, 5, 11, 17, and 24 h post inoculation was stored at −80°C and it was used for RNA extraction and transcriptional analysis of putative catabolic genes. RNA extraction was performed with Nucleospin RNA II kit (Macherey-Nagel, Düren, Germany) followed by a DNAse treatment step (DNAse I, Amplification Grade, Invitrogen Life Technologies). DNA-free RNA was reverse transcribed to obtain cDNA (kit Superscript II, Invitrogen Life Technologies) using random hexamers (Takara, Shiga, Japan).

Primers for the specific amplification of putative catabolic genes identified in the genome of *P. putida* DPA1 and also for the *gyrB* gene (used as reference gene in transcription analysis) were manually designed (Supplementary Table 1) and checked for the formation of secondary structures by the PrimerSelect™ program (Lasergene®, DNASTAR). The specificity of each primer set was assessed *in silico* with the online tool Primer-BLAST (http://www.ncbi.nlm.nih.gov/tools/primer-blast/) and by PCR using DNA of the targeted bacterial strain as a template. The amplicons obtained were checked for the correct size and sequenced to verify amplification of the target gene.

The expression patterns of the target genes were determined via RT-q-PCR with reaction mixtures composed of 5 μl of 2x SYBR Green PCR MasterMix (Kapa, Finland), 20 pmoles of each primer, 1 μl template cDNA and sterile distilled water to a total volume of 10 μl. Thermal conditions were 95°C for 3 min followed by 40 cycles of 95°C for 15 s and 20 s at 56°C. For detection of eventual amplification artifacts a melting-curve analysis was performed immediately after completion of the RT-q-PCR run (95°C for 15 s, 55°C for 30 s, and then slowly increasing the temperature to 95°C). Quantification of gene expression was performed as previously described by Pfaffl (2001). Transcriptional analysis data were subjected to two-way ANOVA and significant differences were detected with the post-hoc Tukey test (*p* < 0.05) using the SPSS Statistics software (IBM Corp. Version 21.0.).

### Plasmid curing of *P. putida* strain DPA1

*Pseudomonas putida* cells were plasmid cured as described by Deshpande et al. (2001) with slight modifications. *Pseudomonas putida* cells were grown overnight at 180 rpm/26°C in LB (NaCl 10 g L^−1^, bacteriological peptone 10 g L^−1^, yeast extract 5 g L^−1^). Aliquots (0.2 ml) from the overnight culture were transferred in 5 ml of fresh LB which was allowed to grow for 2 h at 180 rpm/26°C. At this point ethidium bromide was added in the culture at a concentration of 500 mg L^−1^ and the cultures were left to grow at 180 rpm/40°C for 24 h. An aliquot of the bacterial culture (100 μl) was removed, pelleted by centrifugation and re-suspended in LB which was then plated on fresh LB agar plates. The plates were incubated overnight at 26°C. Fifty colonies growing on the plates were picked up individually and inoculated in fresh LB cultures which were grown overnight at 180 rpm/26°C to allow ample bacterial growth. An aliquot of the bacterial cultures (300 μl) was pelleted by centrifugation, washed twice with sterilized water and re-suspended in MSMN to an OD_600_ of 0.1 before used for the inoculation of MSMN+DPA (25 mg L^−1^). DPA degradation was checked at 7, 14, and 21 days by HPLC analysis. Strains showing no degradation of DPA were further tested for their capacity to degrade aniline and catechol in MSMN (25 mg L^−1^). The degradation of DPA, aniline and catechol by the WT strain was studied in parallel for comparison purposes. For each treatment duplicate inoculated and non-inoculated cultures were prepared.

Strains showing no capacity to degrade DPA and/or aniline were further processed for plasmid extraction and PCR amplification of catabolic genes. Total DNA was extracted from the wild type (WT) and the cured *P. putida* cells using the Purelink Genomic DNA Mini kit (Invitrogen Life Technologies, USA). Plasmid DNA was extracted from WT and plasmid-cured strains as described previously by Turnbull et al. (2001). The presence of selected genes with a putative role in the transformation of DPA (*bphA1A2, tdnA1A2, benA, catA2, pcaD1, pcaD5*) on the plasmid and total DNA of WT and plasmid-cured *P. putida* DPA1 was performed via PCR using the gene specific primers designed for the transcriptional analysis (Supplementary Table 1). Amplifications were carried out in 25 μL reactions containing 1 U of KapaTaq polymerase (Kapa Biosystems, USA), 0.4 μM of each primer, 1x KapaTaqbuffer with Mg^2+^ (with Mg^2+^ at a 1x concentration of 1.5 mM) and 200 μM of each dNTP. The thermal cycling conditions were 95°C for 5 min, followed by 35 cycles of 95°C for 1 min, 56°C for 30 s and 72°C for 30 s, with a final extension of 72°C for 10 min.

## Results

### Genomic analysis of *P. putida*

**Figure 1:**
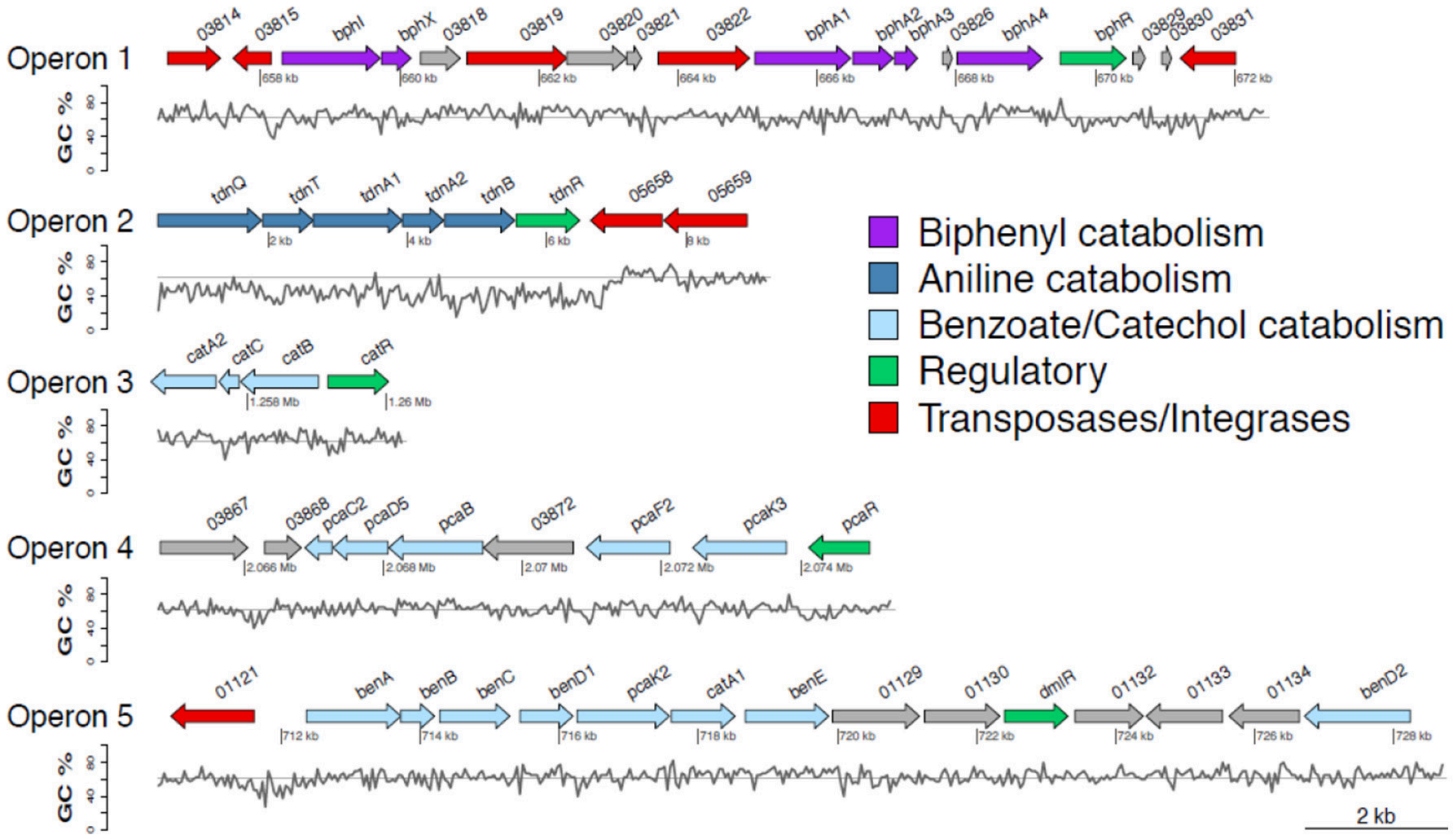
Genetic organization of operons 1–5 containing genes involved in the transformation of diphenylamine (DPA) by *P. putida* strain DPA1. Operon 1 (63.3% GC) is located in scaffold 2 and encodes a multi-component biphenyl dioxygenase with a putative role in the first step of the metabolic pathway of DPA. Operon 2 (47% GC) is located at the 5′ end of scaffold 4 and encodes a multicomponent aniline dioxygenase involved in the transformation of aniline to catechol. Operons 3 (64% GC) and 5 (62.8% GC) are located in scaffold 1. The former encodes a catechol dioxygenase (*catA*) along with *catBC* and the transcriptional regulator *catR* of the *ortho*-cleavage pathway of catechol. The latter encodes an incomplete *ben/cat* operon. Operon 4 (62.4% GC) is located in scaffold 2 and encodes an incomplete protocatechuate cleavage pathway (*pcaCB*) and its oxoadipate branch (*pcaDF*). The %GC variation in the different regions of the catabolic operons 1–5 is also shown; the solid line represents the average GC% content of the genome of *P. putida* DPA1 (62%). ORFs annotation is shown in Table 1.

The draft genome of *P. putida* DPA1 had a total size of 6,266,225 bps with a mean GC content of 62% and a coding density of 74.6% (5679 protein coding genes). It was composed of 91 contigs assembled in 14 scaffolds with the five largest ones having sizes of 3.5, 2.1, 0.6, 0.08, and 0.07 Mbp. Five well organized operons composed of genes with a potential role in the transformation of DPA were detected in scaffolds 1, 2, and 4 (Table 1, Figure 1). Operon 1 (scaffold 2) encoded an incomplete biphenyl transformation pathway (*bph*) composed of (a) *bphA1A2A3A4*, coding for a multi-component biphenyl dioxygenase (b) *bphR*, which encodes a LysR-family transcriptional regulator and (c) *bphI*, coding a 4-hydroxy-2-oxovalerate aldolase, which participates in the lower branch of the *bph* pathway. The GC content of this operon (63.2%) did not vary substantially from the mean GC content of the bacterial genome (Figure 1). Operon 1 was rich in transposases belonging to different insertion sequence (IS)-families (Table 1). They were flanking the operon and located upstream and downstream of the *bphA1A2A3A4R* and *bphIX* regions (Figure 1). Phylogenetic analysis revealed that the proteins encoded by the catabolic genes of operon 1 (indicative BphA1, Supplementary Figure 1a) clustered together with the large subunit of biphenyl-2,3-dioxygenase or benzene-1,2 dioxygenase found in bacteria of the order *Burkholderiales*.

**Table 1:** A list of genes identified in the genome of *P. putida* DPA 1 with a putative role in DPA transformation.

**No**.	**Locus tag**	**Gene**	**Closest homologous protein**	**Assigned function**
**OPERON 1 (BIPHENYL DIOXYGENASE)**				
1	CBL13_03814		Putative transposase (IS91 family)	
2	CBL13_03815		Transposase mutator family (IS256 family)	
3	CBL13_03816	*bphI*	Putative 4-hydroxy-2-oxovalerate aldolase	4-hydroxy-2-oxovalerate aldolase
4	CBL13_03817	*bphX*	bphF 3′-region hypothetical protein Pseudomonas KKS012	
5	CBL13_03818		Hypothetical protein	
6	CBL13_03819		Integrase core domain protein (IS481 family)	
7	CBL13_03820		Hypothetical protein	
8	CBL13_03821		Hypothetical protein	
9	CBL13_03822		Transposase mutator family (IS256 family)	
10	CBL13_03823	*dpaA1*	Biphenyl dioxygenase large subunit	DPA dioxygenase subunit alpha
11	CBL13_03824	*dpaA2*	Biphenyl dioxygenase small subunit	DPA dioxygenase subunit beta
12	CBL13_03825	*dpaA3*	Biphenyl dioxygenase ferredoxin subunit	DPA dioxygenase ferredoxin subunit
13	CBL13_03826		Hypothetical protein	
14	CBL13_03827	*dpaA4*	Anthranilate 1,2-dioxygenase ferredoxin reductase component	DPA dioxygenase ferredoxin reductase component
15	CBL13_03828	*bphR*	HTH-type transcriptional regulator BenM	Transcriptional regulator of the LysR family
16	CBL13_03829		Hypothetical protein	
17	CBL13_03830		Hypothetical protein	
18	CBL13_03831		Transposase (IS21 family)	
**OPERON 2 (ANILINE DIOXYGENASE)**				
19	CBL13_05652	*tdnQ*	Glutamine synthetase	Aniline dioxygenase glutamine synthetase component
20	CBL13_05653	*tdnT*	Glutamine amidotransferase	Aniline dioxygenase glutamine amidotransferase component
21	CBL13_05654	*tdnA1*	2-halobenzoate 1,2-dioxygenase large subunit	Aniline dioxygenase alpha subunit
22	CBL13_05655	*tdnA2*	3-phenylpropionate dioxygenase subunit beta	Aniline dioxygenase beta subunit
23	CBL13_05656	*tdnB*	3-ketosteroid-9-alpha-hydroxylase reductase subunit	Aniline dioxygenase reductase component
24	CBL13_05657	*tdnR*	DNA-binding transcriptional activator GcvA (LysR family)	Aniline dioxygenase transcriptional regulator
25	CBL13_05658		Integrase core domain protein (IS30 family)	
26	CBL13_05659		Transposase mutator family (IS256 family)	
**OPERON 3 (*****CAT*** **PATHWAY)**				
42	CBL13_01888	*catA2*	Catechol 1,2-dioxygenase	Catechol 1,2-dioxygenase
43	CBL13_01889	*catC*	Muconolactone δ-isomerase	Muconolactone δ-isomerase
44	CBL13_01890	*catB*	Muconate cycloisomerase	Muconate cycloisomerase
45	CBL13_01891	*catR*	HTH-type transcriptional regulator BenM	Putative transcriptional regulator LysR family
**OPERON 4 (PROTOCATECHUATE PATHWAY)**				
46	CBL13_03867		Porin-like protein	
47	CBL13_03868		Hypothetical protein	
48	CBL13_03869	*pcaC2*	Carboxymuconolactone decarboxylase family protein	Carboxymuconolactone decarboxylase
49	CBL13_03870	*pcaD5*	3-oxoadipate enol-lactonase	3-oxoadipate enol-lactonase
50	CBL13_03871	*pcaB*	2,3-carboxy-cis-cis-muconate cycloisomerase	2,3-carboxy-cis-cis-muconate cycloisomerase
51	CBL13_03872		Alpha-ketoglutarate permease	
52	CBL13_03873	*pcaF2*	Beta-ketoadipyl-CoA thiolase	Beta-ketoadipyl-CoA thiolase
53	CBL13_03874	*pcaK3*	4-hydroxybenzoate transporter	4-hydroxybenzoate transporter
54	CBL13_03875	*pcaR*	Pca regulon regulatory protein	Pca regulon regulatory protein
**OPERON 5 (*****BEN-CAT*** **PATHWAY)**				
27	CBL13_01121		Transposase mutator family (IS256 family)	
28	CBL13_01122	*benA*	2-halobenzoate 1,2-dioxygenase large subunit	Benzoate dioxygenase large subunit
29	CBL13_01123	*benB*	2-halobenzoate 1,2-dioxygenase small subunit	Benzoate dioxygenase small subunit
30	CBL13_01124	*benC*	Benzoate 1,2-dioxygenase electron transfer component	Benzoate dioxygenase electron transfer component
31	CBL13_01125	*benD1*	Levodione reductase	Benzoate dioxygenase reductase component
32	CBL13_01126	*pcaK2*	4-hydroxybenzoate transporter	
33	CBL13_01127	*catA1*	Catechol 1,2-dioxygenase	Catechol 1,2-dioxygenase
34	CBL13_01128	*benE*	Inner membrane protein	Benzoate membrane transport protein
35	CBL13_01129		Porin-like protein	
36	CBL13_01130		Hypothetical protein	
37	CBL13_01131	*dmlR*	HTH-type transcriptional regulator	Transcriptional regulator LysR family
38	CBL13_01132		Hypothetical protein	
39	CBL13_01133	*nemA*	N-ethylmaleimide reductase	
40	CBL13_01134	*gpr_1*	L-glyceraldehyde 3-phosphate reductase	
41	CBL13_01135	*benD2*	Benzene 1,2-dioxygenase ferredoxinreductase subunit	Benzoate dioxygenase reductase component
**OTHERS PUTATIVE CATABOLIC GENES**				
55	CBL13_00004	*ligB*	Aromatic ring-opening dioxygenase subunit B	
56	CBL13_00557	*ligD*	4-oxalomesaconate tautomerase	
57	CBL13_00558	*ligK*	4-carboxy-4-hydroxy-2-oxoadipic acid aldolase	
58	CBL13_00559	*ligJ*	4-oxalmesaconate hydratase	
59	CBL13_00560	*gbrR*	HTH-type transcriptional regulator	
60	CBL13_00561		Porin-like protein NicP precursor	
61	CBL13_00562	*ligA*	Gallate dioxygenase	
62	CBL13_00563	*pcaK1*	4-hydroxybenzoate transporter	
63	CBL13_00639	*ligC*	4-carboxy-2-hydroxymuconate-6-semialdehyde dehydrogenase	
64	CBL13_00737	*pobA*	p-hydroxybenzoate hydroxylase	
65	CBL13_00933	*pcaC1*	Carboxymuconolactone decarboxylase family protein	
66	CBL13_01046	*pcaI1*	3-oxoadipate CoA-transferase subunit A	
67	CBL13_01047	*pcaJ1*	3-oxoadipate CoA-transferase subunit B	
68	CBL13_01220	*pcaF1*	Beta-ketoadipyl-CoA thiolase	
69	CBL13_01483	*pcaD1*	3-oxoadipate enol-lactonase	
70	CBL13_01858	*pcaD2*	3-oxoadipate enol-lactonase	
71	CBL13_02043	*pcaI2*	3-oxoadipate CoA-transferase subunit A	3-oxoadipate CoA-transferase subunit A
72	CBL13_02044	*pcaJ2*	3-oxoadipate CoA-transferase subunit B	3-oxoadipate CoA-transferase subunit B
73	CBL13_02626	*pcaD3*	3-oxoadipate enol-lactonase	
74	CBL13_02882	*pcaD4*	3-oxoadipate enol-lactonase	
75	CBL13_04043	*pcaG*	Protocatechuate 3,4-dioxygenase alpha chain	
76	CBL13_04044	*pcaH*	Protocatechuate 3,4-dioxygenase beta chain	

Operon 2 (scaffold 4) was composed of genes *tdnQTA1A2BR* encoding a complete aniline dioxygenase (*tdn*) (Figure 1, Table 1). The 3′ end of the operon was also flanked by transposases from different IS families. The operon was characterized by an average GC content of 47%, well below the mean GC content of the bacterial genome (Figure 1, Table 1). Gene organization in operon 2 showed high synteny with the corresponding *tdn/tad* operons of other aniline-degrading strains (Supplementary Figure 2). Phylogenetic analysis revealed that the proteins encoded by operon 2 (indicative TdnQ, Supplementary Figure 1b) clustered together with orthologous proteins belonging to bacteria of the orders *Burkholderiales* and *Sphingomonadales*.

Operons 3 and 5 (scaffold 1) showed GC content (64 and 62.8% respectively) similar to the mean GC content of the bacterial genome (Figure 1). Operon 3 was composed of genes *catA2CBR* encoding the catechol branch (*cat*) of the beta-ketoadipate pathway (Figure 1, Table 1); the *ortho*-cleavage of catechol to 3-oxoadipate enol-lactone. Operon 5 encodes an incomplete benzoate cleavage (*ben*) pathway composed of (a) *benABCD*, encoding a multi-component benzoate dioxygenase plus a second copy of the *benD* gene, (b) *catA1*, encoding a catechol 1,2-dioxygenase, (c) proteins associated with the trans-membrane transportation of benzoate (*benM, pcaK*) and (d) a putative transcriptional regulatory protein of the LysR family *(dmlR)* (Figure 1, Table 1).

Finally operon 4 (scaffold 2) was composed of genes encoding (i) the protocatechuate branch (*pca*) of the beta-ketoadipate pathway (*pcaC2, pcaB, pcaR*) and (ii) the lower section of the beta-ketoadipate pathway (*pcaD5, pcaF2*), which involves the transformation of 3-oxoadipate enol-lactone to Krebs cycle intermediates (Figure 1, Table 1). Its GC content (62.4%) was similar to the mean GC content of the bacterial genome (Figure 1). Four further copies of *pcaD* and two copies of *pcaIJ* were found scattered in scaffold 1, complementing *pcaD5F2* in the lower section of the β-ketoadipate pathway (Table 1). Phylogenetic analysis of the proteins encoded by operons 3, 4 and 5 (CatA, BenA, PcaD, PcaI, Supplementary Figures 1c–f) showed close association with orthologues from other Pseudomonads. Genes involved in different branches of the protocatechuate pathway were also found either organized in operons (i.e., *ligDKJRA* involved in the protocatechuate 4,5-cleavage pathway) or scattered in scaffolds 1 and 2 (*pobA* and *pcaGH* encoding a p-hydroxybenzoate hydroxylase and a protocatechuate 3,4-dioxygenase respectively) (Table 1).

**Figure 2:**
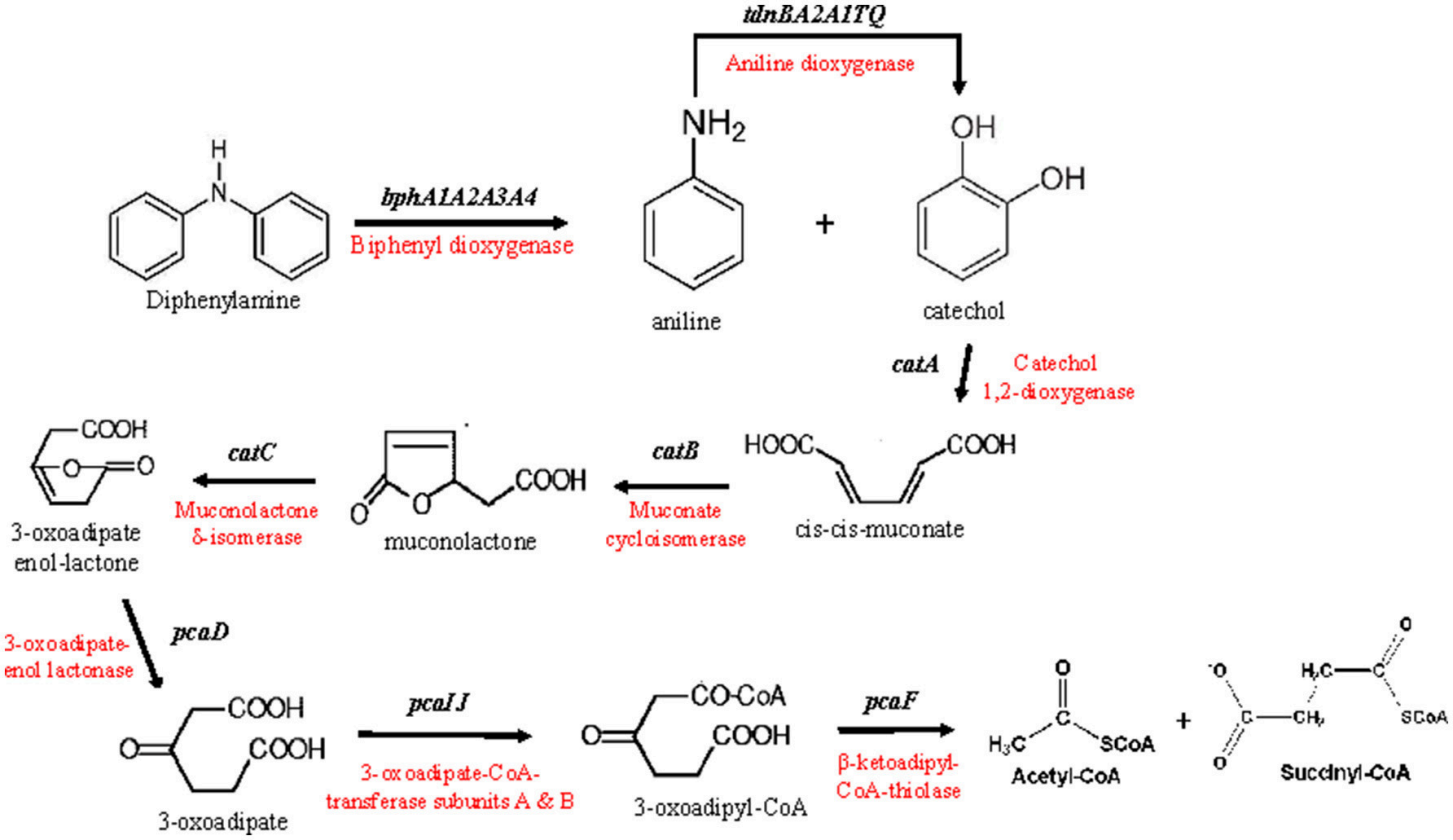
The putative metabolic pathway of diphenylamine by *P. putida* strain DPA1, as depicted by the genomic analysis of the bacterium. The genes and enzymes involved in each step are indicated.

Based on the genomic analysis a putative metabolic pathway of DPA was proposed (Figure 2). DPA is initially transformed to catechol and aniline by the action of the biphenyl dioxygenase *bphA1A2A3A4*. Aniline is subsequently transformed to catechol by the aniline dioxygenase (*tdnQTA1A2BR*). The catechol produced is metabolized via the *ortho*-cleavage pathway, encoded by genes *catABC* and *pcaDIJF*, to Krebs cycle intermediates. The proposed pathway was further validated by proteomics, transcription analysis and plasmid curing assays.

### Proteomic analysis of *P. putida*

**Figure 3:**
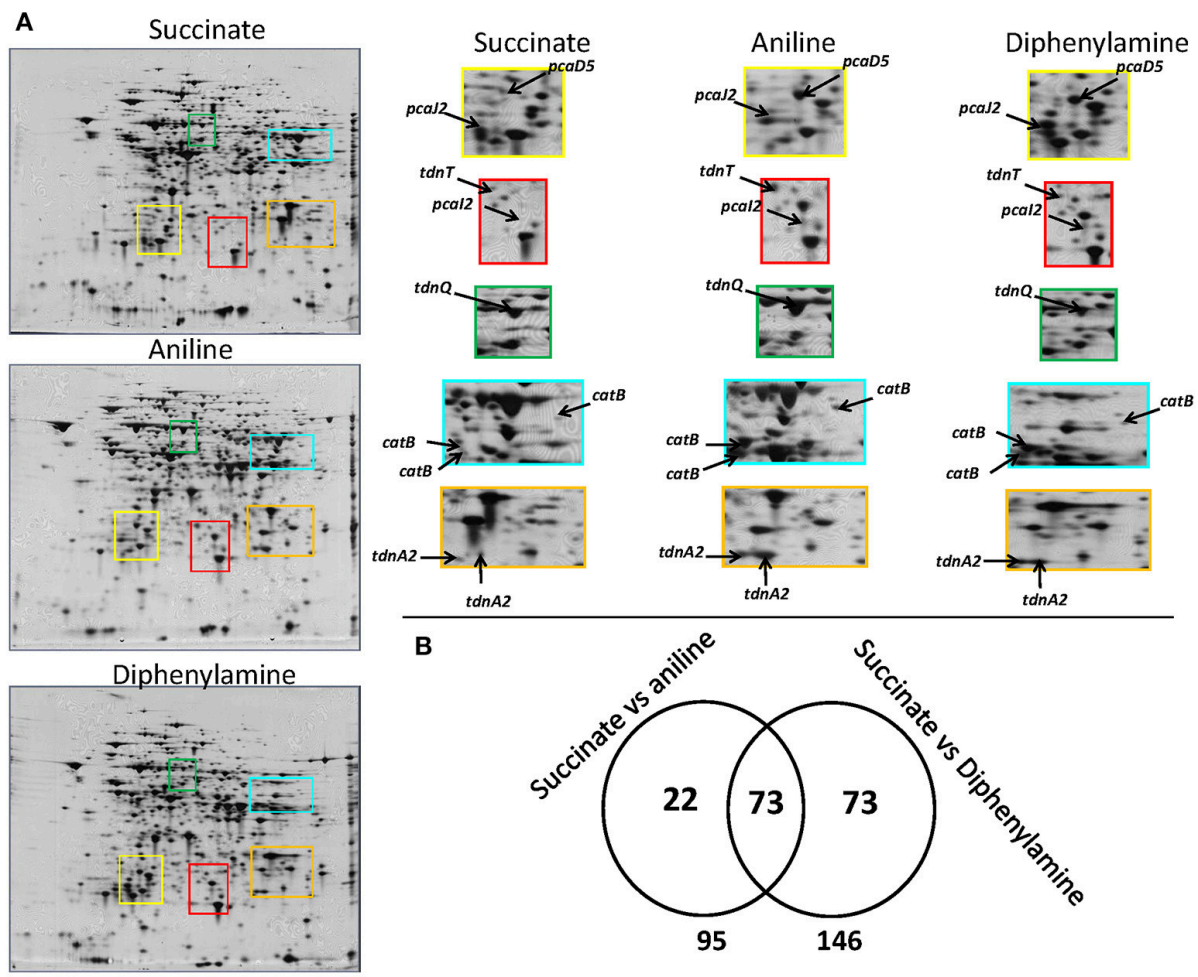
**(A)** 2D gels representing the proteome of *P. putida* cells grown in MSMN supplemented with diphenylamine (DPA), aniline or succinate. Colored frames on the 2D gels are enlarged to focus on the different intensity of spots (associated to selected catabolic proteins) in the different treatments, **(B)** Venn diagram representing the shared and unique protein spots from the aniline- and DPA-growing cells compared to succinate (95 and 146 respectively, with 73 proteins overlapping).

A complete degradation of DPA and aniline was observed within 20 h (Supplementary Figure 3a) and bacterial pellet for protein extraction was collected from all treatments at the late mid-log phase (Supplementary Figure 3b). 2D-gel based proteomic analysis (Figure 3A) identified 168 protein spots which showed differential expression in the DPA and/or aniline treatments compared to succinate (Figure 3B). Amongst these protein spots, 95 and 146, of which 73 shared, showed significantly different expression in the aniline- and DPA-grown cells respectively compared to succinate-grown cells (Figure 3B). Differentially expressed protein spots were excised, sequenced and mapped on the genome of *P. putida* DPA1. Quantitative and sequencing data of the proteins identified in the proteome of *P. putida* DPA1 are given in Supplementary Tables 2, 3 respectively.

In total 24 spots from the proteome of *P. putida* DPA1 were associated with 11 proteins with a putative role in DPA transformation (Table 2). All but one of these proteins were significantly up-regulated in the proteome of cells growing on DPA and aniline and showed homology to translated genes from operons 1, 2, 3, and 4. These proteins were (i) the large subunit BphA1 and the ferredoxin reductase component BphA4 of the biphenyl dioxygenase; the former was the sole catabolic protein which was down-regulated in the presence of DPA and aniline (ii) the glutamine synthetase TdnQ, the glutamine amidotransferase TdnT and the small subunit of aniline dioxygenase TdnA2 (Figure 3A), (iii) the muconate cycloisomerase CatB (Figure 3A) and (iv) the proteins associated with the lower part of the *ortho*-cleavage pathway of catechol (PcaB, PcaD5, PcaF2 in operon 4 and PcaI2J2 in scaffold 1).

**Table 2:** Differentially expressed proteins with a putative role in the catabolism of DPA and aniline (ANI) identified in the proteome of *P. putida* grown on DPA or ANI compared to cells grown on Succinate (Succ).

**Spot no**.	**Protein identification^a^**	**Protein name**	**ANI/Succ**	**DPA/Succ**	**Gene locus code^b^**
521	Biphenyl dioxygenase subunit alpha	BphA1	0.52^**^	0.48^**^	CBL13_3823
7513	Biphenyl dioxygenase subunit alpha	BphA1	0.98	0.27^***^	CBL13_3823
7612	Biphenyl dioxygenase subunit alpha	BphA1	0.78	0.26^***^	CBL13_3823
7613	Biphenyl dioxygenase subunit alpha	BphA1	0.72	0.28^**^	CBL13_3823
7616	Biphenyl dioxygenase subunit alpha	BphA1	0.43^**^	0.44^***^	CBL13_3823
8609	Biphenyl dioxygenase subunit alpha	BphA1	0.54	0.26^*^	CBL13_3823
5409	Biphenyl dioxygenase ferredoxin-NAD(+) reductase component	BphA4	2.39^***^	3.11^***^	CBL13_3827
7413	Biphenyl dioxygenase ferredoxin-NAD(+) reductase component	BphA4	32.85^***^	30.82^***^	CBL13_3827
708	Glutamine synthetase component of aniline dioxygenase	TdnQ	2.28^**^	1.48	CBL13_5652
3608	Glutamine synthetase component of aniline dioxygenase	TdnQ	1.12	7.26^***^	CBL13_5652
3714	Glutamine synthetase component of aniline dioxygenase	TdnQ	1.51^**^	2.48^***^	CBL13_5652
4112	Glutamine amidotransferase component of aniline dioxygenase	TdnT	8.55^*^	1.82	CBL13_5653
7001	Aniline dioxygenase subunit beta	TdnA2	4.35^*^	3.13^*^	CBL13_5655
7002	Aniline dioxygenase subunit beta	TdnA2	6.99^**^	4.72^***^	CBL13_5655
7408	Muconate cycloisomerase	CatB	16.66^***^	16.91^***^	CBL13_1890
7413	Muconate cycloisomerase	CatB	32.85^***^	30.82^***^	CBL13_1890
7415	Muconate cycloisomerase	CatB	7.10^***^	12.28^***^	CBL13_1890
1108	3-oxoadipate enol-lactonase	PcaD5	29.56^***^	17.30^*^	CBL13_3870
5109	3-oxoadipate CoA-transferase subunit A	PcaI2	43.84^*^	43.02^*^	CBL13_2043
1109	3-oxoadipate CoA-transferase subunit B	PcaJ2	23.63^**^	27.54^**^	CBL13_2044
7308	Beta-ketoadipyl-CoA thiolase	PcaF2	1.00	4.05^**^	CBL13_3873
7415	Beta-ketoadipyl-CoA thiolase	PcaF2	7.10^***^	12.28^***^	CBL13_3873
8509	3-carboxy-cis, cis-muconate cycloisomerase	PcaB	34.1^*^	25.48^**^	CBL13_3871

The spot intensity ratios of the ANI/Succ and DPA/Succ are shown as a measure of proteins differential expression (p-value < 0.05, < 0.01, < 0.001 are indicated by *, **, and *** respectively).

a *Protein annotation based on homology with the translated genome of P. putida DPA1*.

b *The locus number of the gene which showed the highest homology with the sequenced protein spot*.

Apart from putative catabolic proteins, several proteins associated with the homeostasis of *P. putida* cells were significantly up-regulated in the presence of DPA and/or aniline (Supplementary Table 4). Stress-related proteins like alkyl hydroperoxide reductase, peroxiredoxin and a small heat shock protein were highly up-regulated in the DPA-growing cells, while a less pronounced up-regulation was evident in the aniline-growing cells. Several proteins associated with the transportation of amino acids (i.e., sulfate binding protein, L-amino acid-binding periplasmic protein AapJ, tricarboxylate transporter family receptor) and membrane permeability and stability (i.e., Leucine-, isoleucine-, valine-, threonine-, and alanine-binding periplasmic proteins, YceI, spermidine/putrescine-binding periplasmic proteins) were several folds up-regulated in the DPA- and aniline-growing cells. Finally, proteins involved in energy production (i.e. isocitrate dehydrogenase [NADP], ATP synthase subunit alfa, aldehyde dehydrogenase B) and in the synthesis of biomolecules (i.e., gamma-glutamyl transpeptidase, 3-isopropylmalate dehydratase small subunit 1, elongation factor Tu) were also significantly up-regulated, mostly in the DPA-growing cells, although some of those proteins like isocitrate dehydrogenase [NADP], aldehyde dehydrogenase B and gamma-glutamyl transpeptidase were up-regulated also in the aniline-growing cells.

### Transcriptional analysis of the catabolic genes

Genomic analysis identified genes with a putative role in the transformation of DPA and proteomic analysis further verified the involvement of their translated products in the transformation of DPA. However the role of other genes, which were identified by the genomic analysis as having a putative role in the transformation pathway but not detected in the proteomic analysis, was not clarified. To this end the transcription profile of all putative catabolic genes during DPA and aniline degradation was determined by RT-q-PCR and their expression levels was compared and contrasted with those in succinate-growing cells.

**Figure 4:**
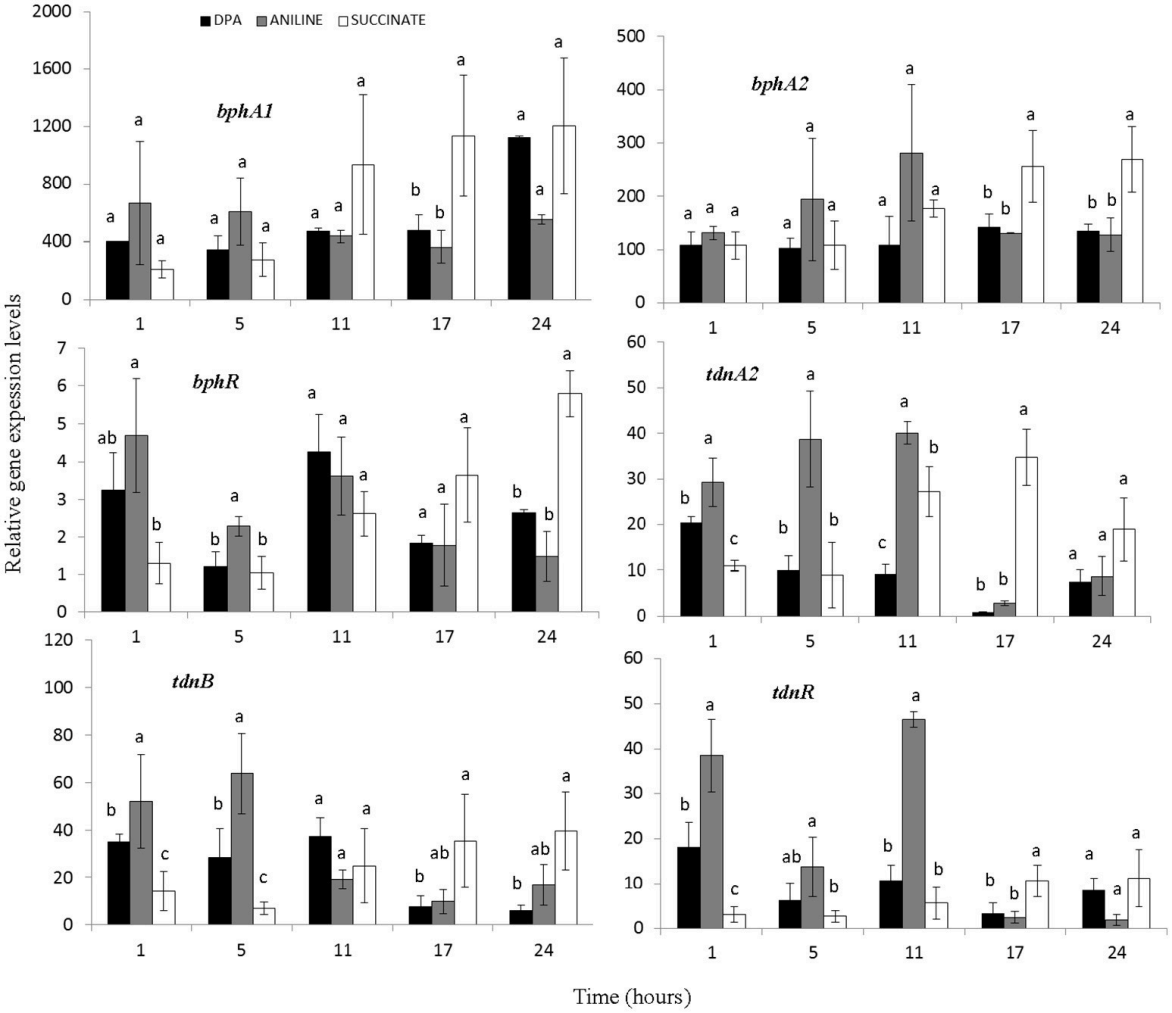
The transcriptional patterns of the *bphA1A2R* (components of the multi-component biphenyl dioxygenase located in operon 1) and *tdnA2BR* (components of the aniline dioxygenase located in operon 2) in cells of the *P. putida* strain DPA1growing in MSMN amended with diphenylamine (DPA), aniline or succinate. Each value is the mean of three replicates ± the standard deviation. Within each time point bars designated by the same letter are not significantly different at the 5% level. The transcription patterns of the other catabolic genes in the *bph* and *tdn* operons 1 and 2 respectively, are shown in Supplementary Figure 4.

All components of the biphenyl dioxygenase (operon 1) and *bphI* showed similar transcription patterns (Figure 4, Supplementary Figure 4). In all treatments particularly high and equivalent expression levels of *bph* genes were observed even 1 h after inoculation. *BphR*, the putative transcriptional regulatory of the biphenyl dioxygenase, showed a significantly higher expression in the presence of aniline during the first 5 h (Figure 4). Genes *tdnQTA1A2BR*, encoding aniline dioxygenase, showed similar transcription patterns (Figure 4, Supplementary Figure 4) with significantly higher expression levels (*p* < 0.05) observed up to 11 h in the presence of aniline compared to DPA and succinate. The expression of aniline dioxygenase genes in the DPA-growing cells was significantly higher compared to succinate in the first 1–5 h.

**Figure 5:**
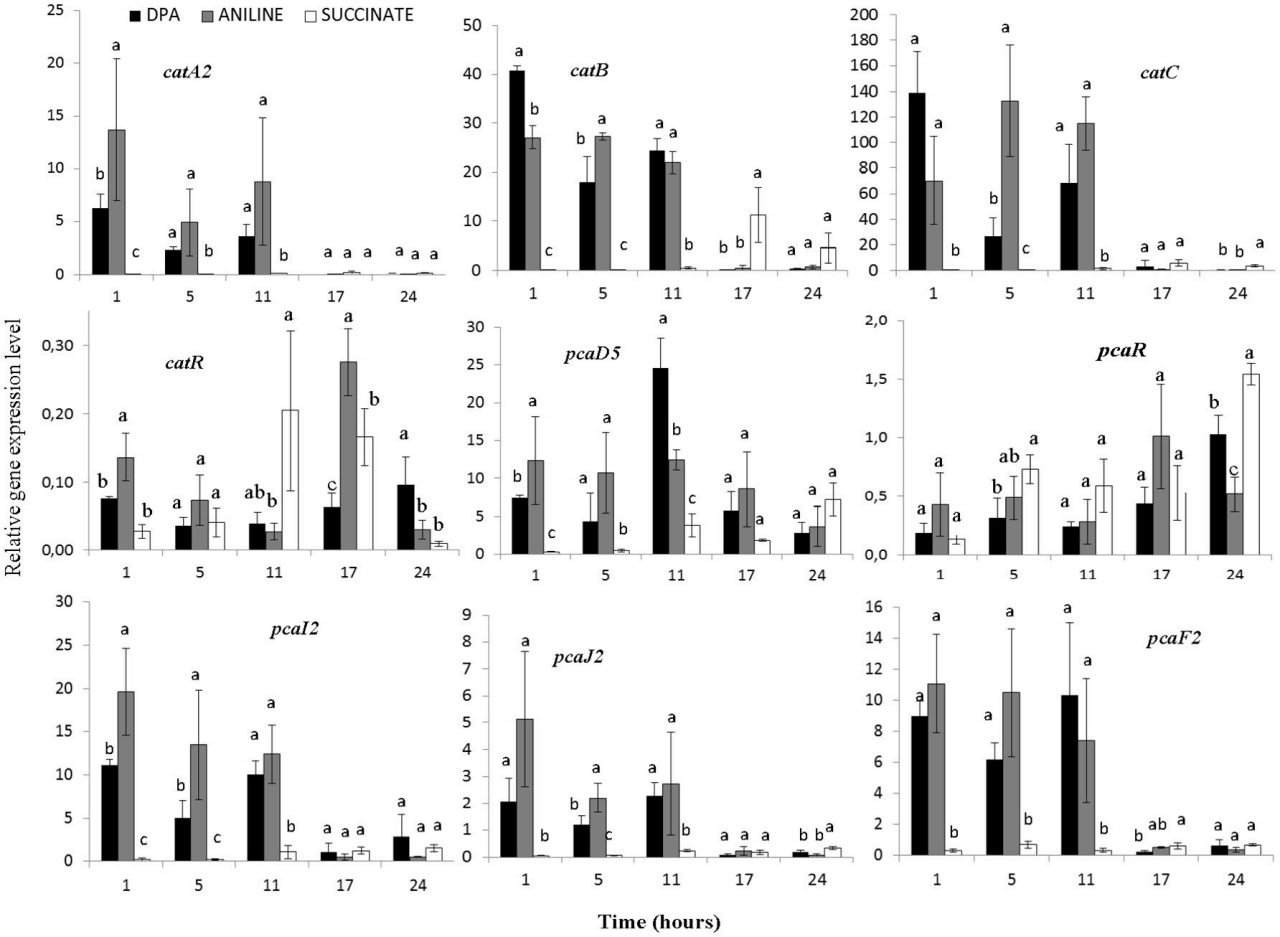
The transcriptional patterns of selected genes of *P. putida* strain DPA1 involved in the transformation of catechol to 3-oxoadipate enol-lactone *(catA2CBR)* and its further transformation to Krebs cycle intermediates (*pcaD5, pcaI2J2, pcaF2, pcaR)*. Cells were grown in MSMN amended with diphenylamine (DPA), aniline or succinate. Within each time point bars designated with the same letter are not significantly different at the 5% level. The transcription patterns of other catabolic with putative role in the *ortho*-cleavage of catechol are given in Supplementary Figures 5, 6.

The transcription profile of genes with potential role in the transformation of catechol was also determined (Figure 5, Supplementary Figures 5–7). Genes *catA2CB* (operon 3) showed significantly higher expression levels (*p* < 0.05) in the presence of aniline and DPA compared to succinate at the first 11 h. Whereas the potential transcriptional regulatory gene of this operon, *catR*, showed low expression levels and its expression pattern did not associate with any of the treatments (Figure 5). In contrast to *catA2*, its orthologue from operon 5 (*catA1*) did not show significant differences (*p* > 0.05) in its expression in the different treatments, in accordance with the transcriptional profile of the rest of the putative catabolic genes of operon 5 (*benABCD1R*) (Supplementary Figure 5).

The transcription patterns of genes involved in the different branches of the protocatechuate pathway organized in operon 4 or found scattered in scaffolds 1 and 2 varied. Genes of operon 4 (*pcaC2D5BF2R*) showed a significant increase in their expression in the presence of aniline and DPA during the first 11 h post inoculation (Figure 5, Supplementary Figure 6). The sole exception was seen in the expression of the putative *pcaR* which did not differ in the different treatments (Supplementary Figure 5). *PcaI2*and *pcaJ2* (scaffold 1), encoding the transformation of 3-oxoadipate to 3-oxoadipyl CoA, showed a significantly higher expression (*p* < 0.05) in the presence of DPA and aniline compared to succinate (Figure 5). Genes *pcaD1, pcaD2*, and *pcaF1* displayed a different expression pattern compared to their orthologues in operon 4 (*pcaD5* and *pcaF2*) with their transcription patterns not significantly differing in the different treatments (Supplementary Figure 6). Finally, *pobA, ligAB*, and *pcaGH* showed similar expression patterns in the different treatments (Supplementary Figure 7).

### Plasmid curing

**Figure 6:**
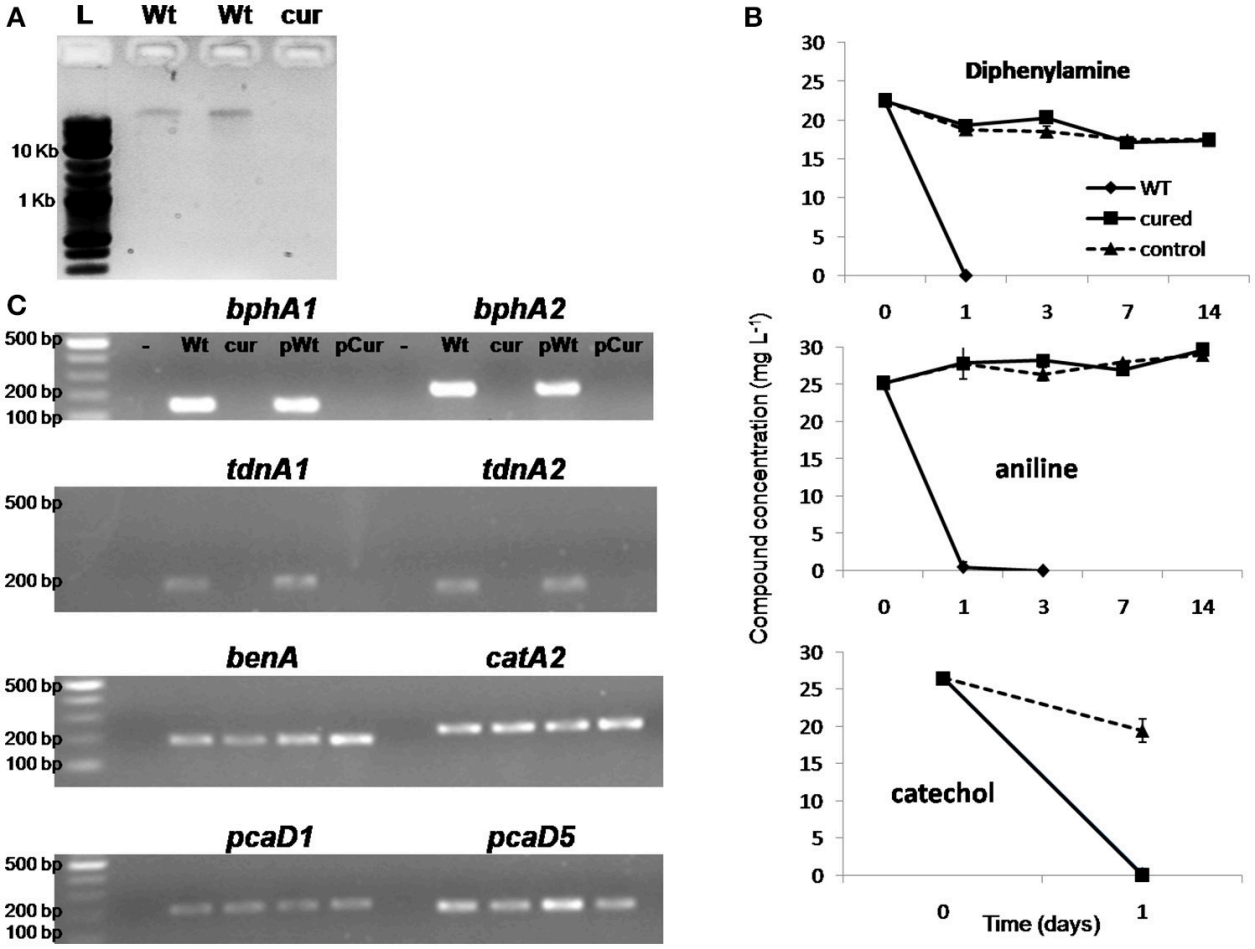
**(A)** Agarose gel electrophoresis (0.8%) showing the plasmid DNA extracted by the wild type strain *P. putida* DPA1 (lanes Wt) and the respective plasmid cured strain; **(B)** The degradation of diphenylamine (DPA), aniline and catechol in MSMN inoculated either with the wild type (WT) or the corresponding plasmid cured strain *P. putida* DPA1. Degradation of the compounds in non-inoculated controls (control) is also shown. Each value is the mean replicates ± the standard deviation of the mean. **(C)** Agarose gel electrophoresis (1.5%) showing PCR detection of genes *bphA1, bphA2, tdnA1, tdnA2, benA, catA2, pcaD1*, and *pcaD5* using as template total (Wt, cur) or plasmid DNA (pWt, pCur) from the Wt or the plasmid cured strain respectively. Negative controls are in the lanes indicated with “-.” The molecular size ladders used are indicated with “L”.

Ethidium bromide treatment of *P. putida* DPA1 resulted in the recovery of one transformant which showed no capacity to degrade DPA. Plasmid profiling of the wild type (WT) and the non-degrading transformant showed the presence of a single plasmid only in the former (Figure 6A). Catechol was rapidly degraded by both plasmid-cured and the WT strain (Figure 6B). However, no degradation of DPA and aniline was evident in 14 days in the cultures inoculated with the plasmid-cured strain, in contrast to the WT which degraded both compounds in 24 h. *BphA1A2* and *tdnA1A2* were successfully amplified only from the DNA, total and plasmid, of the WT strain (Figure 6C). On the contrary, genes with a role in the *ortho*-cleavage pathway of catechol, localized in operons 3, 4, 5 (*benA, catA2*, and *pcaD5*) and scaffold 1 (*pcaD1*), were successfully amplified from the total and plasmid DNA of both the cured and the WT strain.

## Discussion

Genomic analysis of *P. putida* DPA1 revealed a wealth of genes involved in the catabolism of aromatic organic compounds of anthropogenic or natural origin, which were organized in five operons (*bph, tdn, cat, ben*, and *pca*), although a few putative catabolic genes were also found scattered in the draft genome.

The *bph* operon contained a multi-component biphenyl dioxygenase with its transcriptional regulator and remnants of the lower biphenyl pathway (*bphI*). The biphenyl dioxygenase components were disrupted by an ORF of unknown function (CBL13_03826), which resided between *bphA3* and *bphA4*. A similar organization of the components of the biphenyl dioxygenase was observed in *Pseudomonas pseudoalcaligenes* KF707 with the presence of ORF3, a gene of unknown function, between bphA2 and bphA3 (Taira et al., 1992). Biphenyl dioxygenases are ubiquitous in soil bacteria (Furukawa and Fujihara, 2008) and constitute versatile enzymes that initiate the degradation of different aromatic compounds (Furukawa et al., 2004). The large subunit of biphenyl dioxygenase is usually responsible for recognition and binding of substrate, hence a few amino acid changes can lead to significant alterations in its degradation capabilities (Furukawa et al., 2004). In support of this Shindo et al. (2003) obtained a recombinant biphenyl dioxygenase which was able to transform DPA after 4 amino acid changes in the active site of BphA1. All these point to the biphenyl dioxygenase as the enzyme controlling the first step of the DPA pathway and it was designated as a novel DPA dioxygenase (DpaA1A2A3A4). Shin and Spain (2009) identified a carbazole dioxygenase as responsible for initiating the transformation of DPA in *Burkholderia* sp. strain JS667. The latter is a two-component dioxygenase (Ia type) composed of a FNR c-type reductase, a [2Fe-2S] type feredoxin and a single terminal oxygenase component, whereas the biphenyl dioxygenase of *P. putida* DPA1 is a three-component dioxygenase (V type) composed of a GR-type reductase, a [2Fe-2S] type ferredoxin and a terminal oxygenase component with two subunits (Kweon et al., 2008).

Our initial hypothesis concerning DPA dioxygenase was further tested via proteomic and transcription analysis. Proteomics showed that BphA1 and BphA4 were down- and up-regulated respectively in cells growing on DPA, while the expression of the different dioxygenase components was not induced by DPA. However the particularly high expression levels of biphenyl dioxygenase in all treatments, the highest amongst the genes studied, and right from the onset of the experiment, suggests its constitutive expression. This is supported by the lack of lag phase in the degradation of DPA by *P. putida* DPA1 cells growing overnight on glucose (Perruchon et al., 2015), a growth condition which is known to induce carbon catabolic repression in *Pseudomonas* strains (Yuste et al., 1998). A constitutive expression of the DPA-dioxygenase was also evident in the DPA-degrading *Burkholderia* sp. strain JS667 (Shin and Spain, 2009), while in *Pseudomonas* sp. KKS102 disruption of the *bphS* regulatory gene resulted in constitutive expression of *bph* genes (Ohtsubo et al., 2001). *BphR* did not show a transcription pattern relevant to the positive or negative regulation of the *bph* operon in line with the constitutive expression of biphenyl dioxygenase.

A complete *tdn* operon encoding an aniline dioxygenase was present in the draft genome of *P. putida* DPA1. Its gene organization showed high synteny with the aniline-dioxygenase operons found in known aniline-degrading bacteria like the *P. putida* UCC22 (pTDN1) (Fukumori and Saint, 1997), *Delftia tsuruhatensis* AD9 (Liang et al., 2005), *Frauteria* sp. ANA-18 (Murakami et al., 2003) and the relevant operon of the DPA-degrading *Burkholderia* strain JS667 (Shin and Spain, 2009). In contrast to the other aniline-degrading strains, the aniline dioxygenase operon of *P. putida* DPA1 was localized distantly from operons encoding the downstream *ortho*- (Murakami et al., 2003) or *meta*-cleavage (Liang et al., 2005) of catechol. The involvement of the aniline dioxygenase in the DPA transformation pathway was further confirmed by proteomic and transcription analysis. *TdnR* showed an expression profile identical to the structural genes acting as a positive regulator of aniline dioxygenase, as previously reported by Fukumori and Saint (1997).

Regarding the transformation of catechol, produced by aniline and DPA oxidation, several relevant genes were identified in the draft genome of *P. putida* DPA1. A *cat* operon encoded the catechol branch of the beta-ketoadipate pathway. An orthologue of the *catA* gene (*catA1*) was also found in an incomplete *ben* operon next to a multi-component benzoate dioxygenase. In addition the *pobA, ligAB, pcaGH* genes, found scattered in the draft genome of *P. putida* DPA1, and the *pcaC2B*, organized in operon 4, composed an incomplete protocatechuate branch of the beta-ketoadipate pathway. The two branches of the beta-ketoadipate pathway converge to 3-oxoadipate enol-lactone which is further transformed to Krebs cycle intermediates through the action of a common set of enzymes encoded by *pcaDIJF* genes (Li et al., 2010) found in several copies in scaffold 1. The lack of organization of the genes encoding the 3-oxoadipate part of the pathway is a common feature in Pseudomonads (Stover et al., 2000; Paulsen et al., 2005). Functional analysis shed light into the role of the different *cat* and *pca* genes on the transformation of catechol. Proteomics showed a significant up-regulation of (i) catB (operon 3) and pcaB (operon 4), from the catechol and the protocatechuate branches of the beta-ketoadipate pathway respectively, (ii) pcaD5F2 (operon 4) and pcaI2J2 (scaffold 1) encoding the 3-oxoadipate part of the *ortho*-transformation of catechol to Krebs cycle intermediates. Still the role of the two *catA* orthologues in the transformation of catechol was not clarified since no *catA* spots were identified in the proteome of *P. putida* DPA1. Transcription analysis shed light to this and clarified the role of other genes in the transformation of catechol. The expression of the genes of operon 3, including *catA2*, was induced in the presence of DPA and aniline, compared to *catA1* and all the genes localized in operon 5 whose expression was not stimulated by these compounds. These results suggested that operon 3 is driving the *ortho*-cleavage of catechol to 3-oxoadipate enol lactone. Its further transformation is controlled by *pcaD5I2J2F2* genes as verified by the overall stimulation of their transcription in the presence of DPA and aniline. The transcription activation of *pcaBC2* by DPA and aniline is in accordance with the up-regulation of pcaB in the proteome of the corresponding treatments. This is not necessary evidence for a direct involvement of these two genes in the transformation of catechol, but it is probably the result of their co-transcription with the other *pca* genes of operon 4 (*pcaD5, pcaF*) which are directly involved in the transformation of 3-oxoadipate enol-lactone to Krebs cycle intermediates. In support of this is the lack of any transcription activation of the other genes of the protocatechuate branch of the pathway (*pobA, ligAB, pcaGH*) during growth on DPA and aniline.

Both *bph* and *tdn* operons were flanked by transposase suggesting their lateral acquisition by horizontal gene transfer. A further support to this is provided by the phylogenetic affiliation of the proteins encoded by the *bph* and *tdn* genes to phylogenetically distant bacteria. Previous studies have demonstrated the localization of *bph* (Pieper and Seeger, 2008) and *tdn/atd* genes (Takeo et al., 1998) in transposable elements. In contrast to the *bph* operon, which was rich in extraneous elements and transposases, the *tdn* operon showed a compact organization suggesting that its evolution preceded the acquisition of the *bph* operon. We propose that the *P. putida* DPA1 was originally an aniline-degrading bacterium which recently acquired the *bph* operon under the selection pressure exerted in soil by DPA contamination. In contrast operons 3 and 4, encoding the *ortho*-cleavage of catechol, did not contain transposases and their translated products were phylogenetically assigned to the genus *Pseudomonas* suggesting that these genes constitute part of the inherent catabolic network of *P. putida* DPA1 for the degradation of biogenic aromatic compounds thriving in the soil environment (Udaondo et al., 2016).

*Pseudomonas putida* DPA1 appeared to carry a plasmid whose size was not determined. Plasmid curing resulted in loss of *bph* and *tdn* genes and its degrading ability for DPA and aniline. However the cured strain was still able to transform catechol and maintained the genes associated with its transformation. Pseudomonads are known to carry plasmids encoding xenobiotics transformation pathways (Ma et al., 2006), channeling such compounds to metabolites that feed into central metabolic pathways (Timmis, 2002; Fernández et al., 2012). These results further support our initial suggestion that the *bph* and *tdn* operons are part of transposable elements which were acquired by *P. putida* DPA1 through horizontal gene transfer and reinforce the central role of the biphenyl dioxygenase in the degradation of DPA by *P. putida* DPA1.

Overall our findings suggest that the metabolic pathway of DPA in *P. putida* DPA1 is composed of three sections: (a) DPA is initially oxidized to catechol and aniline (b) the latter is further oxidized to catechol which (c) is finally transformed to acetyl-CoA and succinyl-CoA through the *ortho*-cleavage pathway. The proposed pathway is in line with Perruchon et al. (2015), which reported the transient formation of aniline and catechol during DPA degradation *P. putida* DPA1. However it deviates from the pathway reported for *Burkholderia* sp. strain JS667 where the catechol formed was transformed via the *meta*-cleavage pathway (Shin and Spain, 2009).

In parallel to the study of the metabolic network of *P. putida* DPA1, proteomic analysis provided a wider view of the overall response of the bacterial cells to DPA and aniline. *P. putida* cells growing on DPA, and secondly on aniline, showed an up-regulation of proteins like alkyl hydroperoxide reductase (Seaver and Imlay, 2001), peroxiredoxin (Dubbs and Mongkolsuk, 2007), Dps (Martinez and Kolter, 1997), and IdpB (Kitagawa et al., 2000) known to be associated with oxidative stress response mechanisms in bacteria. Alkyl hydroperoxide reductase was identified as one of the most up-regulated proteins in the cells of *P. putida* KT2440 growing on different aromatic compounds (Kim et al., 2006). Several membrane transporters and proteins involved in membrane stability were also up-regulated in DPA and aniline treatments, in line with previous proteomic studies in bacteria exposed to organic pollutants (Zhang et al., 2014). Upon exposure to stress environmental conditions microorganisms are known to adjust the fluidity of their cellular membrane by altering their phospholipid composition, driven by *cis/trans* isomerization of unsaturated fatty acids (Nikodinovic-Runic et al., 2009; Mujahid et al., 2015). Leucine, isoleucine and valine are believed to control the ratio of *cis/trans* isomerisation (Vandera et al., 2015), in line with the consistent up-regulation of these amino acid periplasmic binding proteins in the DPA- and aniline-growing cells. Several proteins involved in energy production and in the synthesis of biomolecules were up-regulated, mainly in the presence of DPA. A similar up-regulation of such proteins was reported in a *Stenotrophomonas maltophilia* growing on 17β-estradiol (Li et al., 2012). This can be also considered as part of the general stress-response mechanism activated in *P. putida* DPA1 for the *de novo* synthesis of biomolecules to counterbalance the toxicity of DPA and aniline and the parallel increase in needs of the growing cells. This overall alerting of the stress response mechanism of *P. putida* DPA1 during degradation of DPA and aniline suggests that these compounds are not preferred substrates for the bacterium.

## Conclusions

A step-wise genomic, proteomic and transcription analysis was utilized to dissect the genetic metabolic network of *P. putida* DPA1 associated with the transformation of DPA. A biphenyl dioxygenase, identified as a novel DPA dioxygenase, was responsible for the transformation of DPA to aniline and catechol. The former was transformed by an aniline dioxygenase to catechol, which was further metabolized to Krebs cycle intermediates via the *ortho*-cleavage pathway. The genes of the upper pathway resided most probably in plasmid-encoded transposons suggesting lateral acquisition by horizontal gene transfer. Proteomics revealed the mobilization of a multi-layered stress response mechanism by *P. putida* DPA1 during degradation of DPA and aniline.

## Author contributions

EP was involved in the proteomic experiment and performed the transcription analysis. CP isolated the strain, performed plasmid curing and she was involved in the transcription analysis and contributed to the manuscript writing. SV performed the genome assembly, annotation and the phylogenetic analysis of the catabolic proteins. CR performed the proteomic experiment. GT, MS, and AM were involved in the proteomic analysis of the bacterium. DK had the idea, planned the work and wrote with CP the manuscript which was reviewed by all authors.

### Conflict of interest statement

The authors declare that the research was conducted in the absence of any commercial or financial relationships that could be construed as a potential conflict of interest.

## References

[B1] Abascal F. Zardoya R. Posada D. (2005). ProtTest: selection of best-fit models of protein evolution. Bioinformatics 21, 2104–2105. 10.1093/bioinformatics/bti263 15647292

[B2] Ainalidou A. Tanou G. Belghazi M. Samiotaki M. Diamantidis G. Molassiotis A.. (2016). Integrated analysis of metabolites and proteins reveal aspects of the tissue-specific function of synthetic cytokinin in kiwifruit development and ripening. J. Proteomics 143, 318–333. 10.1016/j.jprot.2016.02.013 26915585

[B3] Altschul S. F. Gish W. Miller W. Myers E. W. Lipman D. J. (1990). Basic local alignment search tool. J. Mol. Biol. 215, 403–410. 10.1016/S0022-2836(05)80360-2 2231712

[B4] Bers K. Leroy B. Breugelmans P. Albers P. Lavigne R. Sørensen S. R.. (2011). A novel hydrolase identified by genomic-proteomic analysis of phenylurea herbicide mineralization by *Variovorax* sp strain SRS16. Appl. Environ. Microbiol. 77, 8754–8764. 10.1128/AEM.06162-11 22003008 PMC3233098

[B5] Bradford M. M. (1976). A rapid and sensitive method for the quantitation of microgram quantities of protein utilizing the principle of protein-dye binding. Anal. Biochem. 72, 248–254. 10.1016/0003-2697(76)90527-3 942051

[B6] Deshpande N. M. Dhakephalkar P. K. Kanekar P. P. (2001). Plasmid-mediated dimethoate degradationin *Pseudomonas aeruginosa* MCMB-427. Lett. Appl. Microbiol. 33, 275–279. 10.1046/j.1472-765X.2001.00995.x 11559400

[B7] Drzyzga O. (2003). Diphenylamine and derivatives in the environment: a review. Chemosphere 53, 809–818. 10.1016/S0045-6535(03)00613-1 14505701

[B8] Dubbs J. M. Mongkolsuk S. (2007). Peroxiredoxins in bacterial antioxidant defense. Subcell. Biochem. 44, 143–193. 10.1007/978-1-4020-6051-9_7 18084893

[B9] Edgar R. C. (2004). MUSCLE: a multiple sequence alignment method with reduced time and space complexity. BMC Bioinform. 5:113. 10.1186/1471-2105-5-113 15318951 PMC517706

[B10] Entenmann W. Schacke G. (1994). The wide-spread diffusive contamination of a former military airport from propellant contamination: investigations and consequences for the establishment of development plants, in Abstract retrieved from Luxembourg International Symposium on the Rehabilitation of Former Military Sites and Demilitarization of Explosive Ordinance (Luxembourg City), 405–415.

[B11] European Food Safety Authority (EFSA) (2012). Conclusion on the peer review of the pesticide risk assessment of the active substance diphenylamine. EFSA J. 10, 2486–2545. 10.2903/j.efsa.2012.2486

[B12] Fernández M. Niqui-Arroyo J. L. Conde S. Ramos J. L. Duque E. (2012). Enhanced tolerance to naphthalene and enhanced rhizoremediation performance for *Pseudomonas putida* KT2440 via the NAH7 catabolic plasmid. Appl. Environ. Microbiol. 78, 5104–5110. 10.1128/AEM.00619-12 22582075 PMC3416403

[B13] Fukumori F. Saint C. P. (1997). Nucleotide sequences and regulational analysis of genesinvolved in conversion of aniline to catechol in *Pseudomonas putida* UCC22 (pTDN1). J. Bacteriol. 179, 399–408. 10.1128/jb.179.2.399-408.1997 8990291 PMC178709

[B14] Furukawa K. Fujihara H. (2008). Microbial degradation of polychlorinated biphenyls: biochemical and molecular features. J. Biosci. Bioeng. 105, 433–449. 10.1263/jbb.105.433 18558332

[B15] Furukawa K. Suenaga H. Goto M. (2004). Biphenyl dioxygenases: functional versatilities and directed evolution. J. Bacteriol. 186, 5189–5196. 10.1128/JB.186.16.5189-5196.2004 15292119 PMC490896

[B16] Gunasekera T. S. Bowen L. L. Zhou C. E. Howard-Byerly S. C. Foley W. S. Striebich R. C.. (2017). Transcriptomic analyses elucidate adaptive differences of closely related strains of *Pseudomonas aeruginosa* in fuel. Appl. Environ. Microbiol. 83, e03249–e03216. 10.1128/AEM.03249-16 28314727 PMC5411511

[B17] Guy L. Kultima J. R. Andersson S. G. (2010). GenoPlotR: comparative gene and genome visualization in R. Bioinformatics 26, 2334–2335. 10.1093/bioinformatics/btq413 20624783 PMC2935412

[B18] Kim Y. H. Cho K. Yun S. H. Kim J. Y. Kwon K. H. Yoo J. S.. (2006). Analysis of aromatic catabolic pathways in *Pseudomonas putida* KT 2440 using a combined proteomic approach: 2-DE/MS and cleavable isotope-coded affinity tag analysis. Proteomics 6, 1301–1318. 10.1002/pmic.200500329 16470664

[B19] Kitagawa M. Matsumura Y. Tsuchido T. (2000). Small heat shock proteins, IbpA and IbpB, are involved in resistances to heat and superoxide stresses in *Escherichia coli*. FEMS Microbiol. Lett. 184, 165–171. 10.1111/j.1574-6968.2000.tb09009.x 10713416

[B20] Kweon O. Kim S. J. Baek S. Chae J. C. Adjei M. D. Baek D. H.. (2008). A new classification system for bacterial Rieske non-heme iron aromatic ring-hydroxylating oxygenases. BMC Biochem. 9:11. 10.1186/1471-2091-9-11 18387195 PMC2358900

[B21] Li D. Yan Y. Ping S. Chen M. Zhang W. Li L. (2010). Genome-wide investigation and functional characterization of the b-ketoadipate pathway inthe nitrogen-fixing and root-associated bacterium *Pseudomonas stutzeri* A1501. BMC Microbiol. 10, 36–49. 10.1186/1471-2180-10-36 20137101 PMC2907835

[B22] Li Z. Nandakumar R. Madayiputhiya N. Li X. (2012). Proteomic analysis of 17β-Estradiol degradation by *Stenotrophomonas maltophilia*. Environ. Sci. Technol. 46, 5947–5955. 10.1021/es300273k 22587609

[B23] Liang Q. Takeo M. Chen M. Zhang W. Xu Y. Lin M. (2005). Chromosome-encoded gene cluster for the metabolic pathway that converts aniline to TCA-cycle intermediates in *Delftia tsuruhatensis* AD9. Microbiology 151, 3435–3446. 10.1099/mic.0.28137-0 16207925

[B24] Lye J. Freeman H. S. (1999). Azo- and nitro-diphenylamine dye photochemistry. Adv. Colour Sci. Technol. 2, 112–119.

[B25] Ma Y. Wang L. Shao Z. (2006). *Pseudomonas*, the dominant polycyclic aromatic hydrocarbon-degrading bacteria isolated from Antarctic soils and the role of large plasmids in horizontal gene transfer. Environ. Microbiol. 8, 455–465. 10.1111/j.1462-2920.2005.00911.x 16478452

[B26] Martinez A. Kolter R. (1997). Protection of DNA during oxidative stress by the nonspecific DNA-binding protein Dps. J. Bacteriol. 179, 5188–5194. 10.1128/jb.179.16.5188-5194.1997 9260963 PMC179379

[B27] Masubuchi Y. Yamada S. Horie T. (1999). Diphenylamine as an important structure of nonsteroidal anti-inflammatory drugs to uncouple mitochondrial oxidative phosphorylation. Biochem. Pharmacol. 58, 861–865. 10.1016/S0006-2952(99)00163-X 10449197

[B28] Mujahid M. Prasuna M. L. Sasikala C. Ramana C. V. (2015). Integrated metabolomic and proteomic analysis reveals systemic responses of *Rubrivivax benzoatilyticus* JA2 to aniline stress. J. Proteome Res. 14, 711–727. 10.1021/pr500725b 25388363

[B29] Murakami S. Hayashi T. Maeda T. Takenaka S. Aoki K. (2003). Cloning and functional analysis of aniline dioxygenase gene cluster, from *Frateuria* species ANA-18, that metabolizes aniline via an *ortho*-cleavage pathway of catechol. Biosci. Biotechnol. Biochem. 67, 2351–2358. 10.1271/bbb.67.2351 14646193

[B30] Nikodinovic-Runic J. Flanagan M. Hume A. R. Cagney G. O'Connor K. E. (2009). Analysis of the *Pseudomonas putida* CA-3 proteome during growth on styrene under nitrogen-limiting and non-limiting conditions. Microbiology 155, 3348–3361. 10.1099/mic.0.031153-0 19608612

[B31] Ohtsubo Y. Delawary M. Kimbara K. Takagi M. Ohta A. Nagata Y. (2001). BphS, a key transcriptional regulator of *bph* genes involved in polychlorinated biphenyl/biphenyl degradation in *Pseudomonas* sp. KKS102. J. Biol. Chem. 276, 36146–36154. 10.1074/jbc.M100302200 11459836

[B32] Parks D. H. Imelfort M. Skennerton C. T. Hugenholtz P. Tyson G. W. (2015). CheckM: assessing the quality of microbial genomes recovered from isolates, single cells, and metagenomes. Genome Res. 25, 1043–1055. 10.1101/gr.186072.114 25977477 PMC4484387

[B33] Paulsen I. T. Press C. M. Ravel J. Kobayashi D. Y. Myers G. S. Mavrodi D. V.. (2005). Complete genome sequence of the plant commensal *Pseudomonas fluorescens* Pf-5. Nat. Biotechnol. 23, 873–878. 10.1038/nbt1110 15980861 PMC7416659

[B34] Perruchon C. Batianis C. Zouborlis S. Papadopoulou E. S. Ntougias S. Vasileiadis S.. (2015). Isolation of a diphenylamine-degrading bacterium and characterization of its metabolic capacities, bioremediation and bioaugmentation potential. Environ. Sci. Pollut. Res. 22, 19485–19496. 10.1007/s11356-015-5132-0 26260839

[B35] Perruchon C. Vasileiadis S. Papadopoulou E. S. Rousidou C. Tanou G. Samiotaki M. (2017). Metabolic pathway, cell adaptation mechanisms and a novel monoxygenaseare revealed through proteogenomic-transcription analysis of a Sphingomonas haloaromaticamans strain degrading the fungicide ortho-phenylphenol. Sci. Rep. 7:6449 10.1038/s41598-017-06727-6 28743883 PMC5527002

[B36] Pfaffl M. W. (2001). A new mathematical model for relative quantification in real-time RT-PCR. Nucleic Acids Res. 29:e45. 10.1093/nar/29.9.e45 11328886 PMC55695

[B37] Pieper D. H. Seeger M. (2008). Bacterial metabolism of polychlorinated biphenyls. J. Mol. Microbiol. Biotechnol. 15, 121–138. 10.1159/000121325 18685266

[B38] R Core Team (2015). R: A Language and Environment for Statistical Computing, Reference Index Version 3.2.2. R Foundation for Statistical Computing Available online at: http://www.R-project.org/

[B39] Ribeiro F. J. Przybylski D. Yin S. Sharpe T. Gnerre S. Abouelleil A.. (2012). Finished bacterial genomes from shotgun sequence data. Genome Res. 22, 2270–2277. 10.1101/gr.141515.112 22829535 PMC3483556

[B40] Rudell D. R. Mattheis J. P. Fellman J. K. (2005). Relationship of superficial scald development and alpha-farnesene oxidation to reactions of diphenylamine and diphenylamine derivatives in Cv. Granny Smith apple peel. J. Agric. Food. Chem. 53, 8382–8389. 10.1021/jf0512407 16218691

[B41] Seaver L. C. Imlay J. A. (2001). Alkyl hydroperoxide reductase is the primary scavenger of endogenous hydrogen peroxide in *Escherichia coli*. J. Bacteriol. 183, 7173–7181. 10.1128/JB.183.24.7173-7181.2001 11717276 PMC95566

[B42] Seemann T. (2014). Prokka: rapid prokaryotic genome annotation. Bioinformatics 30, 2068–2069. 10.1093/bioinformatics/btu153 24642063

[B43] Shin K. A. Spain J. C. (2009). Pathway and evolutionary implications of diphenylamine biodegradation by *Burkholderia* sp. strain JS667. Appl. Environ. Microbiol. 75, 2694–2704. 10.1128/AEM.02198-08 19251893 PMC2681709

[B44] Shindo K. Nakamura R. Chinda I. Ohnishi Y. Horinouchi S. Takahashi H. (2003). Hydroxylation of ionized aromatics including carboxylic acid or amine using recombinant *Streptomyces lividans* cells expressing modified biphenyl dioxygenase genes. Tetrahedron 59, 1895–1900. 10.1016/S0040-4020(03)00180-7

[B45] Stamatakis A. (2014). RAxML version 8: a tool for phylogenetic analysis and post-analysis of large phylogenies. Bioinformatics 30, 1312–1313. 10.1093/bioinformatics/btu033 24451623 PMC3998144

[B46] Stover C. K. Pham X. Q. Erwin A. L. Mizoguchi S. D. Warrener P. Hickey M. J. (2000). Complete genome sequence of *Pseudomonas aeruginosa* PA01, an opportunistic pathogen. Nature 406, 959–964. 10.1038/35023079 10984043

[B47] Taira K. Hirose J. Hayashida S. Furukawa K. (1992). Analysis of bph operon from the polychlorinated biphenyl-degrading strain of *Pseudomonas pseudoalcaligenes* KF707. J. Biol. Chem. 267, 4844–4853. 1537863

[B48] Takeo M. Fujii T. Maeda Y. (1998). Sequence analysis of the genes encoding a multicomponent dioxygenase involved in oxidation of aniline and o-toluidine in *Acinetobacter* sp. strain YAA. J. Ferment. Bioengin. 85, 17–24. 10.1016/S0922-338X(97)80347-9

[B49] Talavera G. Castresana J. (2007). Improvement of phylogenies after removing divergent and ambiguously aligned blocks from protein sequence alignments. Syst. Biol. 56, 564–577. 10.1080/10635150701472164 17654362

[B50] Timmis K. (2002). *Pseudomonas putida*: a cosmopolitan opportunist *par excellence*. Environ. Microbiol. 4, 779–781. 10.1046/j.1462-2920.2002.00365.x 12534460

[B51] Trivedi V. D. Jangir P. K. Sharma R. Phale P. S. (2016). Insights into functional and evolutionary analysis of carbaryl metabolic pathway from *Pseudomonas* sp. strain C5pp. Sci. Rep. 6:38430. 10.1038/srep38430 27924916 PMC5141477

[B52] Tsaboula A. Papadakis E. N. Vryzas Z. Kotopoulou A. Kintzikoglou K. Papadopoulou-Mourkidou E. (2016). Environmental and human risk hierarchy of pesticides: a prioritization method, based on monitoring, hazard assessment and environmental fate. Environ. Intern. 91, 78–93. 10.1016/j.envint.2016.02.008 26915710

[B53] Turnbull G. A. Ousley M. Walker A. Shaw E. Morgan J. A. (2001). Degradation of substituted phenylurea herbicides by *Arthrobacter globiformis* strain D47 and characterization of a plasmid-associated hydrolase gene, *puhA*. Appl. Environ. Microbiol. 67, 2270–2275. 10.1128/AEM.67.5.2270-2275.2001 11319111 PMC92866

[B54] Udaondo Z. Molina L. Segura A. Duque E. Ramos J. L. (2016). Analysis of the core genome and pangenome of *Pseudomonas putida*. Environ. Microbiol. 18, 3268–3283. 10.1111/1462-2920.13015 26261031

[B55] Vandera E. Samiotaki M. Parapouli M. Panayotou G. Koukkou A. I. (2015). Comparative proteomic analysis of *Arthrobacter phenanthrenivorans* Sphe3 on phenanthrene, phthalate and glucose. J. Proteom. 113, 73–89. 10.1016/j.jprot.2014.08.018 25257624

[B56] Vilchez-Vargas R. Junca H. Pieper D. H. (2010). Metabolic networks, microbial ecology and 'omics' technologies: towards understanding in situ biodegradation processes. Environ. Microbiol. 12, 3089–3104. 10.1111/j.1462-2920.2010.02340.x 20860734

[B57] Yan X. Gu T. Yi Z. Huang J. Liu X. Zhang J.. (2016). Comparative genomic analysis of isoproturon mineralizing sphingomonads reveals the isoproturon catabolic mechanism. Environ. Microbiol. 18, 4888–4906. 10.1111/1462-2920.13413 27317932

[B58] Yuste L. Canosa I. Rojo F. (1998). Carbon-source-dependent expression of the PalkB promoter from the *Pseudomonas oleovorans* alkane degradation pathway. J. Bacteriol. 180, 5218–5226. 9748457 10.1128/jb.180.19.5218-5226.1998 PMC107560

[B59] Zhang H. Jiang X. Xiao W. Lu L. (2014). Proteomic strategy for the analysis of the polychlorobiphenyl-degrading cyanobacterium *Anabaena* PD-1 exposed to aroclor 1254. PLoS ONE 9:e91162. 10.1371/journal.pone.0091162 24618583 PMC3949748

[B60] Ziogas V. Tanou G. Belghazi M. Filippou P. Fotopoulos V. Diamantidis G.. (2015). Roles of sodium hydrosulfide and sodium nitroprusside as priming molecules during drought acclimation in citrus plants. Plant Mol. Biol. 89, 433–450. 10.1007/s11103-015-0379-x 26404728

