# Supplementary material for: Metabolic and Evolutionary Insights in the Transformation of Diphenylamine by a Pseudomonas putida Strain Unravelled by Genomic, Proteomic, and Transcription Analysis
Source: Front Microbiol. 2018 Apr 6;9:676. doi: 10.3389/fmicb.2018.00676 (PMC5897751; doi:10.3389/fmicb.2018.00676)
Supplement: Supplementary file 1 [file DataSheet1.pdf]

Supplementary Material

Metabolic and evolutionary insights in the transformation of diphenylamine by a *Pseudomonas putida* strain unravelled by genomic, proteomic and transcription analysis

Evangelia S. Papadopoulou,[1+] Chiara Perruchon,[1+] Sotirios Vasileiadis,[1] Constantina Rousidou,[1] Georgia Tanou,[2] Martina Samiotaki,[3] Athanassios Molassiotis,[2] Dimitrios G. Karpouzas [1*]

[1] Laboratory of Plant and Environmental Biotechnology, Department of Biochemistry and Biotechnology, University of Thessaly, Larissa, Greece
[2] School of Agriculture, Aristotle University of Thessaloniki, Thessaloniki, Greece
[3] Biomedical Sciences Research Center "Alexander Fleming", Vari, Greece

[+] **Equal first authors**

*** Corresponding Author**
Dr. Dimitrios G. Karpouzas
dkarpouzas@bio.uth.gr

1. Supplementary Figures and Tables

1.1. **Supplementary Figures**

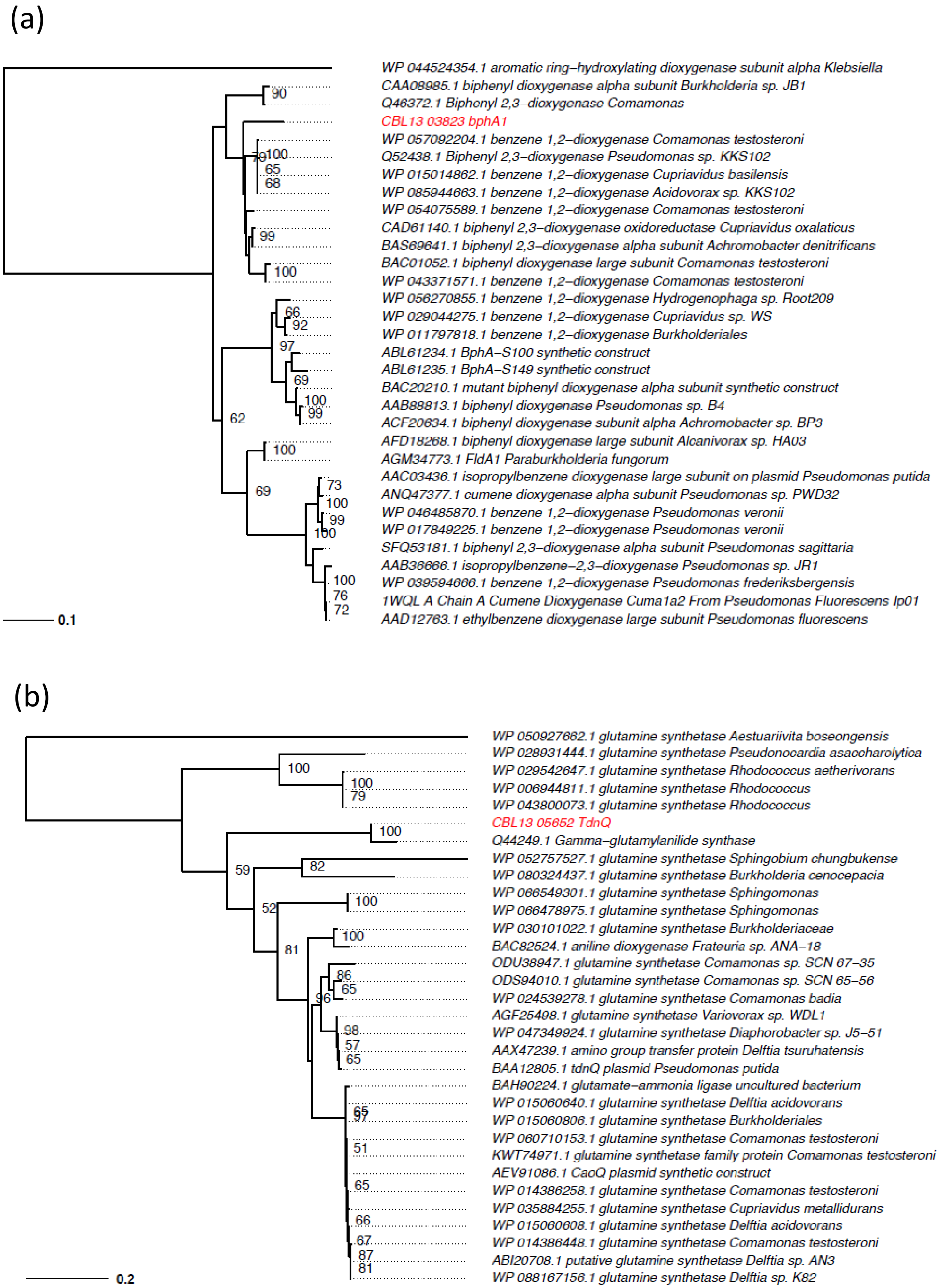

(a)
WP 044524354.1 aromatic ring–hydroxylating dioxygenase subunit alpha Klebsiella
CAA08985.1 biphenyl dioxygenase alpha subunit Burkholderia sp. JB1
Q46372.1 Biphenyl 2,3–dioxygenase Comamonas
CBL13 03823 bphA1
WP 057092204.1 benzene 1,2–dioxygenase Comamonas testosteroni
Q52438.1 Biphenyl 2,3–dioxygenase Pseudomonas sp. KKS102
WP 015014862.1 benzene 1,2–dioxygenase Cupriavidus basilensis
WP 085944663.1 benzene 1,2–dioxygenase Acidovorax sp. KKS102
WP 054075589.1 benzene 1,2–dioxygenase Comamonas testosteroni
CAD61140.1 biphenyl 2,3–dioxygenase oxidoreductase Cupriavidus oxalaticus
BAS69641.1 biphenyl 2,3–dioxygenase alpha subunit Achromobacter denitrificans
BAC01052.1 biphenyl dioxygenase large subunit Comamonas testosteroni
WP 043371571.1 benzene 1,2–dioxygenase Comamonas testosteroni
WP 056270855.1 benzene 1,2–dioxygenase Hydrogenophaga sp. Root209
WP 029044275.1 benzene 1,2–dioxygenase Cupriavidus sp. WS
WP 011797818.1 benzene 1,2–dioxygenase Burkholderiales
ABL61234.1 BphA–S100 synthetic construct
ABL61235.1 BphA–S149 synthetic construct
BAC20210.1 mutant biphenyl dioxygenase alpha subunit synthetic construct
AAB88813.1 biphenyl dioxygenase Pseudomonas sp. B4
ACF20634.1 biphenyl dioxygenase subunit alpha Achromobacter sp. BP3
AFD18268.1 biphenyl dioxygenase large subunit Alcanivorax sp. HA03
AGM34773.1 FldA1 Paraburkholderia fungorum
AAC03436.1 isopropylbenzene dioxygenase large subunit on plasmid Pseudomonas putida
ANQ47377.1 cumene dioxygenase alpha subunit Pseudomonas sp. PWD32
WP 046485870.1 benzene 1,2–dioxygenase Pseudomonas veronii
WP 017849225.1 benzene 1,2–dioxygenase Pseudomonas veronii
SFQ53181.1 biphenyl 2,3–dioxygenase alpha subunit Pseudomonas sagittaria
AAB36666.1 isopropylbenzene–2,3–dioxygenase Pseudomonas sp. JR1
WP 039594666.1 benzene 1,2–dioxygenase Pseudomonas frederiksbergensis
1WQL A Chain A Cumene Dioxygenase Cuma1a2 From Pseudomonas Fluorescens Ip01
AAD12763.1 ethylbenzene dioxygenase large subunit Pseudomonas fluorescens
0.1
(b)
WP 050927662.1 glutamine synthetase Aestuariivita boseongensis
WP 028931444.1 glutamine synthetase Pseudonocardia asaccharolytica
WP 029542647.1 glutamine synthetase Rhodococcus aetherivorans
WP 006944811.1 glutamine synthetase Rhodococcus
WP 043800073.1 glutamine synthetase Rhodococcus
CBL13 05652 TdnQ
Q44249.1 Gamma–glutamylanilide synthase
WP 052757527.1 glutamine synthetase Sphingobium chungbukense
WP 080324437.1 glutamine synthetase Burkholderia cenocepacia
WP 066549301.1 glutamine synthetase Sphingomonas
WP 066478975.1 glutamine synthetase Sphingomonas
WP 030101022.1 glutamine synthetase Burkholderiaceae
BAC82524.1 aniline dioxygenase Frateuria sp. ANA–18
ODU38947.1 glutamine synthetase Comamonas sp. SCN 67–35
ODS94010.1 glutamine synthetase Comamonas sp. SCN 65–56
WP 024539278.1 glutamine synthetase Comamonas badia
AGF25498.1 glutamine synthetase Variovorax sp. WDL1
WP 047349924.1 glutamine synthetase Diaphorobacter sp. J5–51
AAX47239.1 amino group transfer protein Delftia tsuruhatensis
BAA12805.1 tdnQ plasmid Pseudomonas putida
BAH90224.1 glutamate–ammonia ligase uncultured bacterium
WP 015060640.1 glutamine synthetase Delftia acidovorans
WP 015060806.1 glutamine synthetase Burkholderiales
WP 060710153.1 glutamine synthetase Comamonas testosteroni
KWT74971.1 glutamine synthetase family protein Comamonas testosteroni
AEV91086.1 CaoQ plasmid synthetic construct
WP 014386258.1 glutamine synthetase Comamonas testosteroni
WP 035884255.1 glutamine synthetase Cupriavidus metallidurans
WP 015060608.1 glutamine synthetase Delftia acidovorans
WP 014386448.1 glutamine synthetase Comamonas testosteroni
ABI20708.1 putative glutamine synthetase Delftia sp. AN3
WP 088167156.1 glutamine synthetase Delftia sp. K82
0.2

(c)

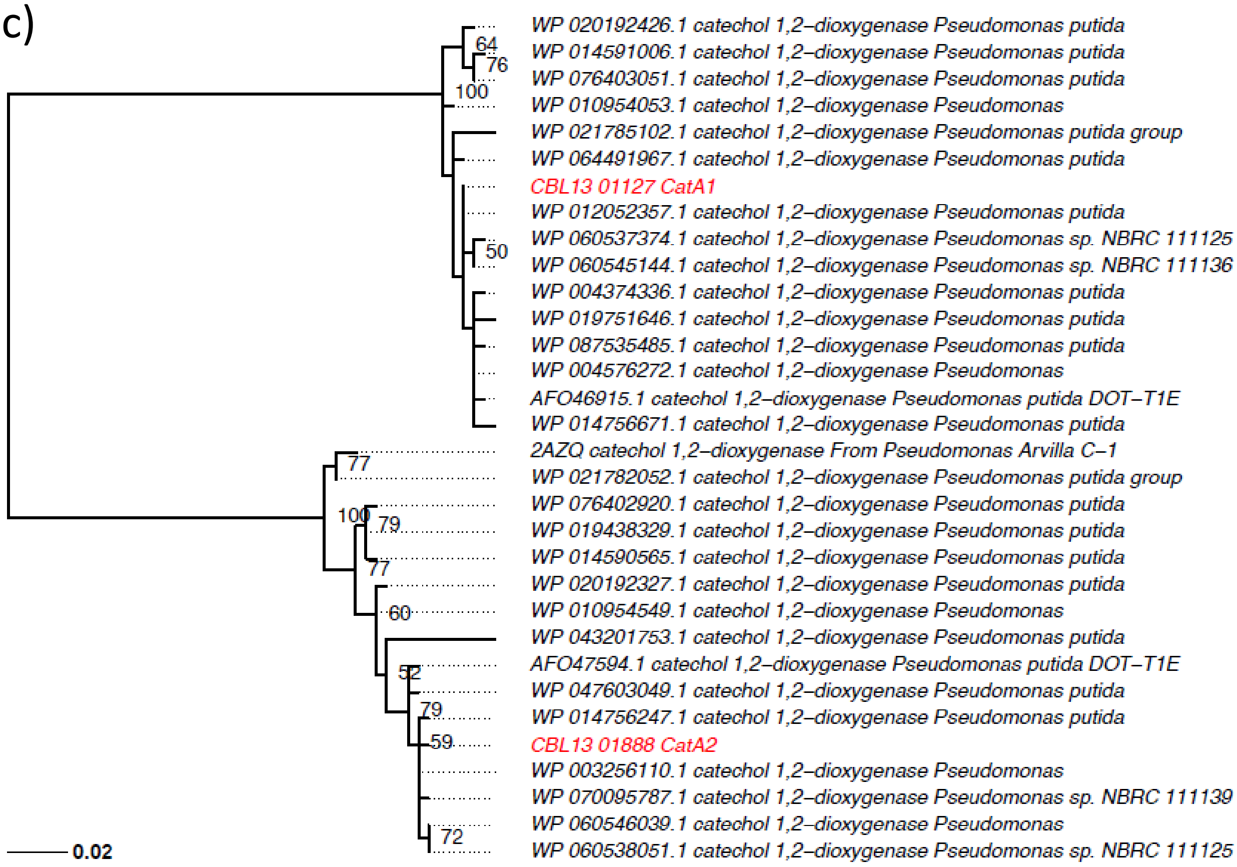
WP 020192426.1 catechol 1,2–dioxygenase Pseudomonas putida
WP 014591006.1 catechol 1,2–dioxygenase Pseudomonas putida
WP 076403051.1 catechol 1,2–dioxygenase Pseudomonas putida
WP 010954053.1 catechol 1,2–dioxygenase Pseudomonas
WP 021785102.1 catechol 1,2–dioxygenase Pseudomonas putida group
WP 064491967.1 catechol 1,2–dioxygenase Pseudomonas putida
CBL13 01127 CatA1
WP 012052357.1 catechol 1,2–dioxygenase Pseudomonas putida
WP 060537374.1 catechol 1,2–dioxygenase Pseudomonas sp. NBRC 111125
WP 060545144.1 catechol 1,2–dioxygenase Pseudomonas sp. NBRC 111136
WP 004374336.1 catechol 1,2–dioxygenase Pseudomonas putida
WP 019751646.1 catechol 1,2–dioxygenase Pseudomonas putida
WP 087535485.1 catechol 1,2–dioxygenase Pseudomonas putida
WP 004576272.1 catechol 1,2–dioxygenase Pseudomonas
AFO46915.1 catechol 1,2–dioxygenase Pseudomonas putida DOT–T1E
WP 014756671.1 catechol 1,2–dioxygenase Pseudomonas putida
2AZQ catechol 1,2–dioxygenase From Pseudomonas Arvilla C–1
WP 021782052.1 catechol 1,2–dioxygenase Pseudomonas putida group
WP 076402920.1 catechol 1,2–dioxygenase Pseudomonas putida
WP 019438329.1 catechol 1,2–dioxygenase Pseudomonas putida
WP 014590565.1 catechol 1,2–dioxygenase Pseudomonas putida
WP 020192327.1 catechol 1,2–dioxygenase Pseudomonas putida
WP 010954549.1 catechol 1,2–dioxygenase Pseudomonas
WP 043201753.1 catechol 1,2–dioxygenase Pseudomonas putida
AFO47594.1 catechol 1,2–dioxygenase Pseudomonas putida DOT–T1E
WP 047603049.1 catechol 1,2–dioxygenase Pseudomonas putida
WP 014756247.1 catechol 1,2–dioxygenase Pseudomonas putida
CBL13 01888 CatA2
WP 003256110.1 catechol 1,2–dioxygenase Pseudomonas
WP 070095787.1 catechol 1,2–dioxygenase Pseudomonas sp. NBRC 111139
WP 060546039.1 catechol 1,2–dioxygenase Pseudomonas
WP 060538051.1 catechol 1,2–dioxygenase Pseudomonas sp. NBRC 111125
0.02

(d)

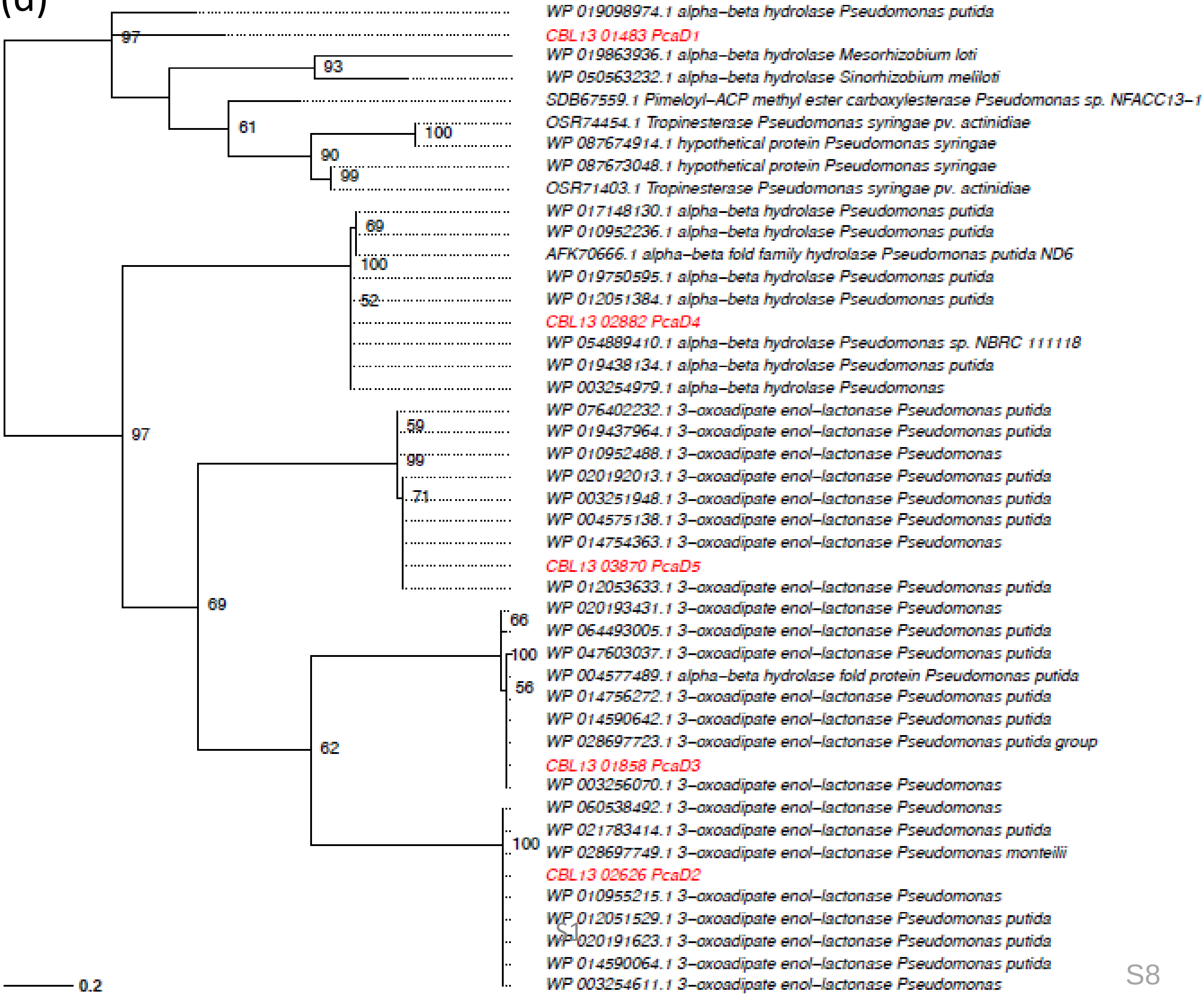
WP 019098974.1 alpha–beta hydrolase Pseudomonas putida
CBL13 01483 PcaD1
WP 019863936.1 alpha–beta hydrolase Mesorhizobium loti
WP 050563232.1 alpha–beta hydrolase Sinorhizobium meliloti
SDB67559.1 Pimeloyl–ACP methyl ester carboxylesterase Pseudomonas sp. NFACC13–1
OSR74454.1 Tropinesterase Pseudomonas syringae pv. actinidiae
WP 087674914.1 hypothetical protein Pseudomonas syringae
WP 087673048.1 hypothetical protein Pseudomonas syringae
OSR71403.1 Tropinesterase Pseudomonas syringae pv. actinidiae
WP 017148130.1 alpha–beta hydrolase Pseudomonas putida
WP 010952236.1 alpha–beta hydrolase Pseudomonas putida
AFK70666.1 alpha–beta fold family hydrolase Pseudomonas putida ND6
WP 019750595.1 alpha–beta hydrolase Pseudomonas putida
WP 012051384.1 alpha–beta hydrolase Pseudomonas putida
CBL13 02882 PcaD4
WP 054889410.1 alpha–beta hydrolase Pseudomonas sp. NBRC 111118
WP 019438134.1 alpha–beta hydrolase Pseudomonas putida
WP 003254979.1 alpha–beta hydrolase Pseudomonas
WP 076402232.1 3–oxoadipate enol–lactonase Pseudomonas putida
WP 019437964.1 3–oxoadipate enol–lactonase Pseudomonas putida
WP 010952488.1 3–oxoadipate enol–lactonase Pseudomonas
WP 020192013.1 3–oxoadipate enol–lactonase Pseudomonas putida
WP 003251948.1 3–oxoadipate enol–lactonase Pseudomonas putida
WP 004575138.1 3–oxoadipate enol–lactonase Pseudomonas putida
WP 014754363.1 3–oxoadipate enol–lactonase Pseudomonas
CBL13 03870 PcaD5
WP 012053633.1 3–oxoadipate enol–lactonase Pseudomonas putida
WP 020193431.1 3–oxoadipate enol–lactonase Pseudomonas
WP 064493005.1 3–oxoadipate enol–lactonase Pseudomonas putida
WP 047603037.1 3–oxoadipate enol–lactonase Pseudomonas putida
WP 004577489.1 alpha–beta hydrolase fold protein Pseudomonas putida
WP 014756272.1 3–oxoadipate enol–lactonase Pseudomonas putida
WP 014590642.1 3–oxoadipate enol–lactonase Pseudomonas putida
WP 028697723.1 3–oxoadipate enol–lactonase Pseudomonas putida group
CBL13 01858 PcaD3
WP 003256070.1 3–oxoadipate enol–lactonase Pseudomonas
WP 060538492.1 3–oxoadipate enol–lactonase Pseudomonas
WP 021783414.1 3–oxoadipate enol–lactonase Pseudomonas putida
WP 028697749.1 3–oxoadipate enol–lactonase Pseudomonas monteilii
CBL13 02626 PcaD2
WP 010955215.1 3–oxoadipate enol–lactonase Pseudomonas
WP 012051529.1 3–oxoadipate enol–lactonase Pseudomonas putida
WP 020191623.1 3–oxoadipate enol–lactonase Pseudomonas putida
WP 014590064.1 3–oxoadipate enol–lactonase Pseudomonas putida
WP 003254611.1 3–oxoadipate enol–lactonase Pseudomonas
0.2

(e)

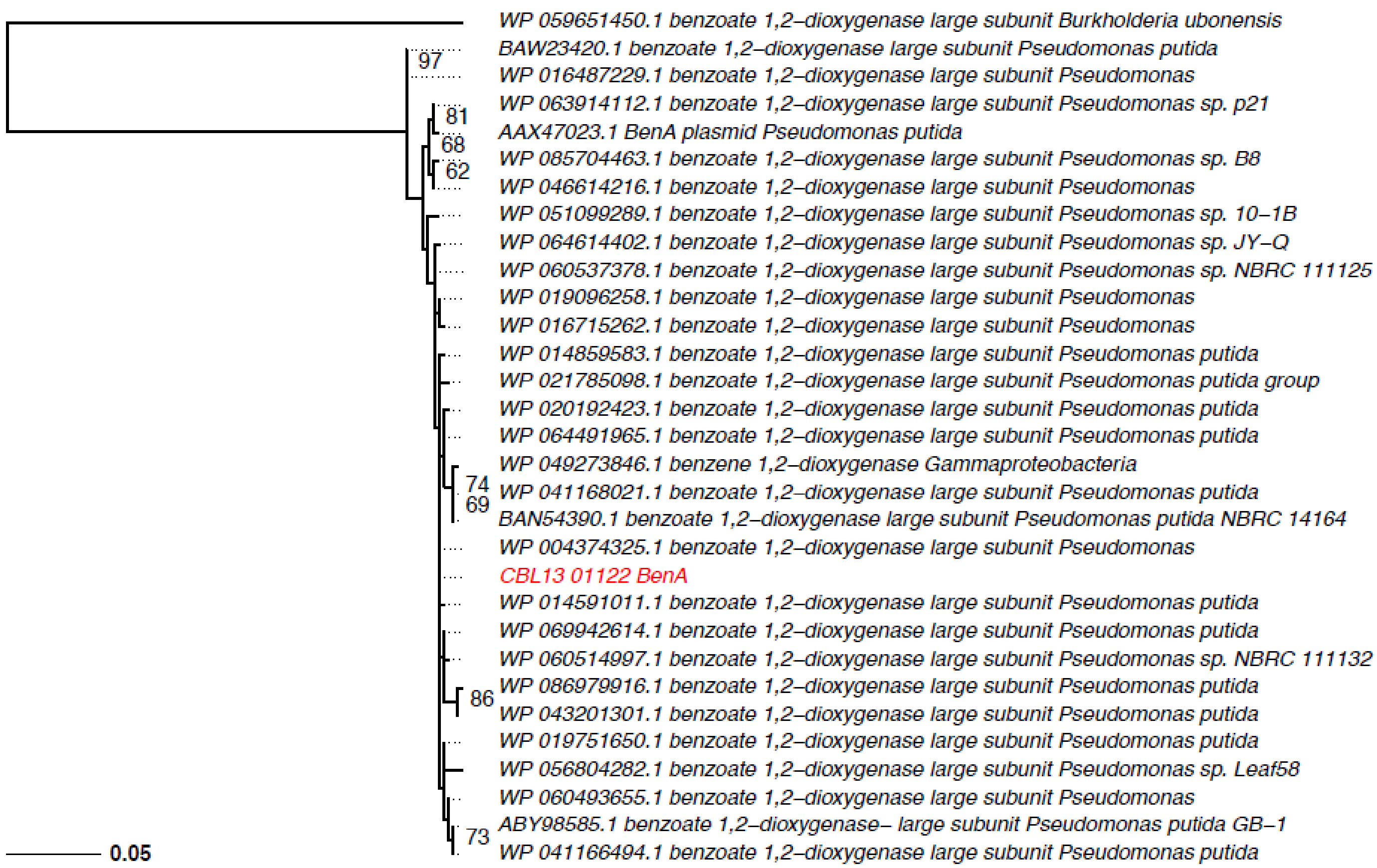

(f)

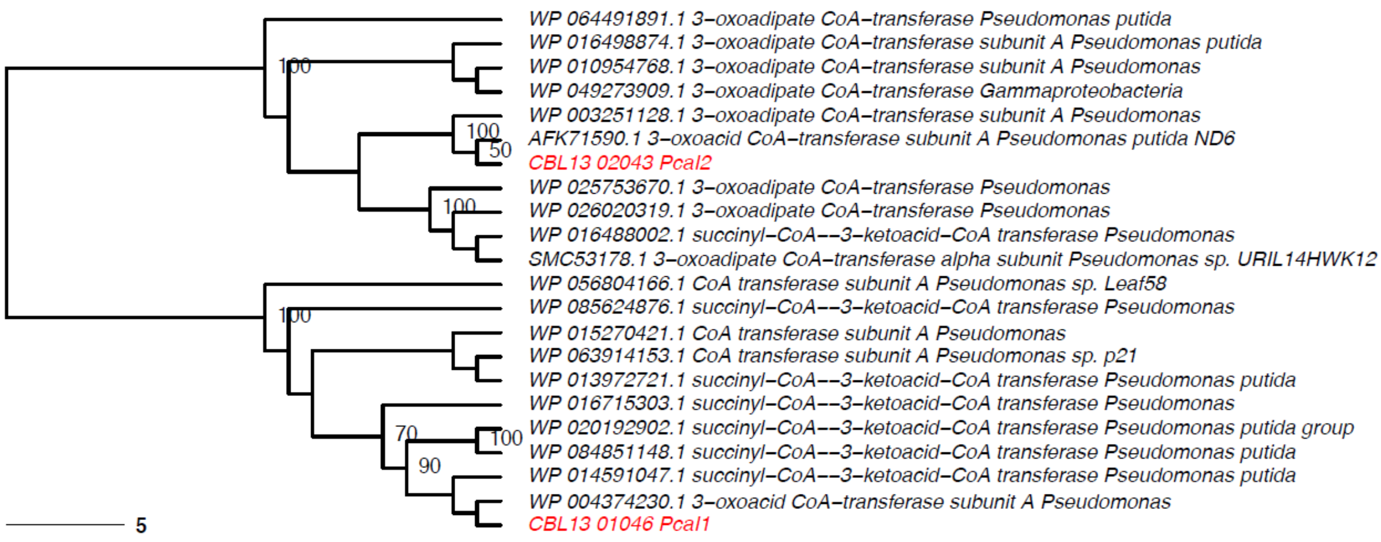

Supplementary Figure 1. Phylogenetic analysis of selected proteins with a putative catabolic role in the transformation of diphenylamine by *P. putida* strain DPA1 (a) BphA1, (b) TdnQ, (c) CatA, (d) PcaD (e) BenA and (f) PcaI. It should be noted that for CatA, PcaD and PcaI, where multiple copies of the corresponding genes were found in the genome of *P. putida* strain DPA1, trees contain all orthologues.

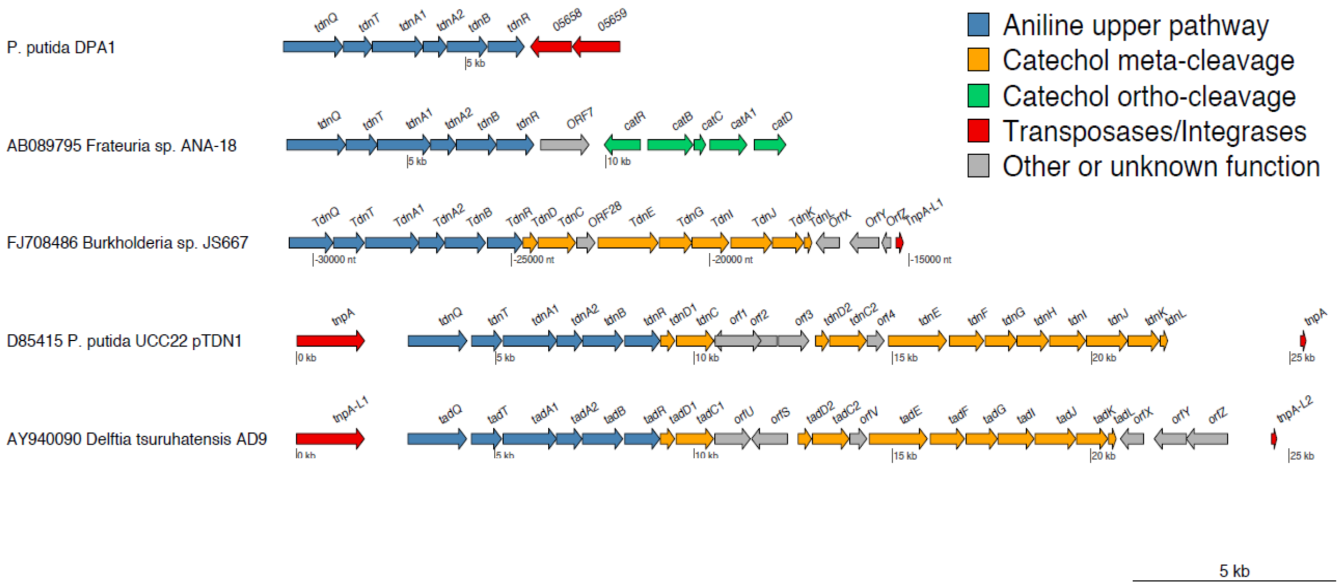

Supplementary Figure 2. A comparative analysis of the organization of the aniline dioxygenase operon (tdn operon 2) in *P. putida* strain DPA1 and in other aniline-degrading strains

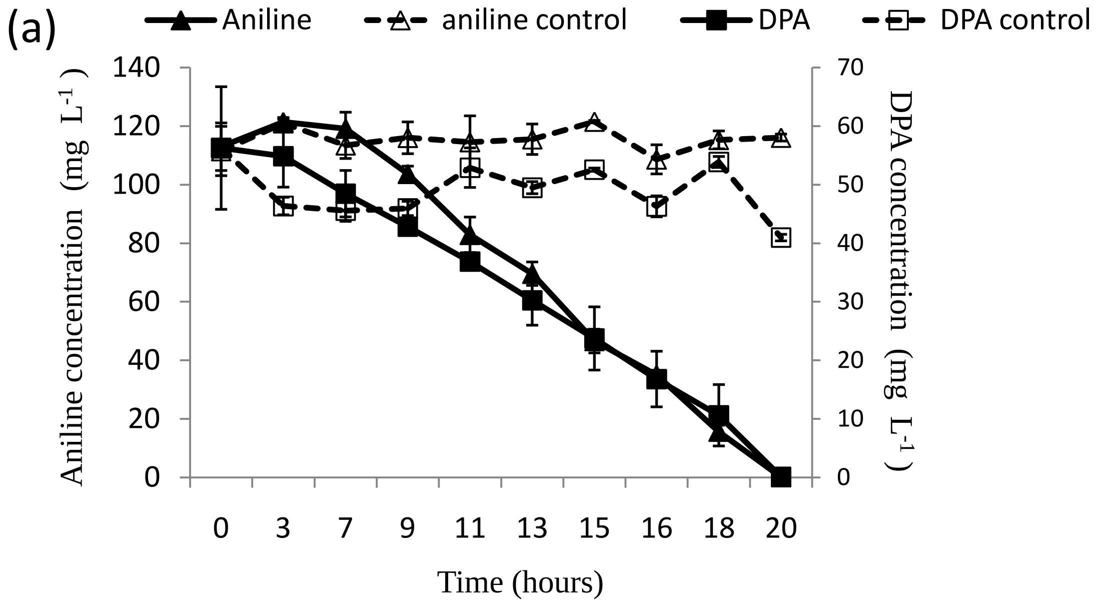

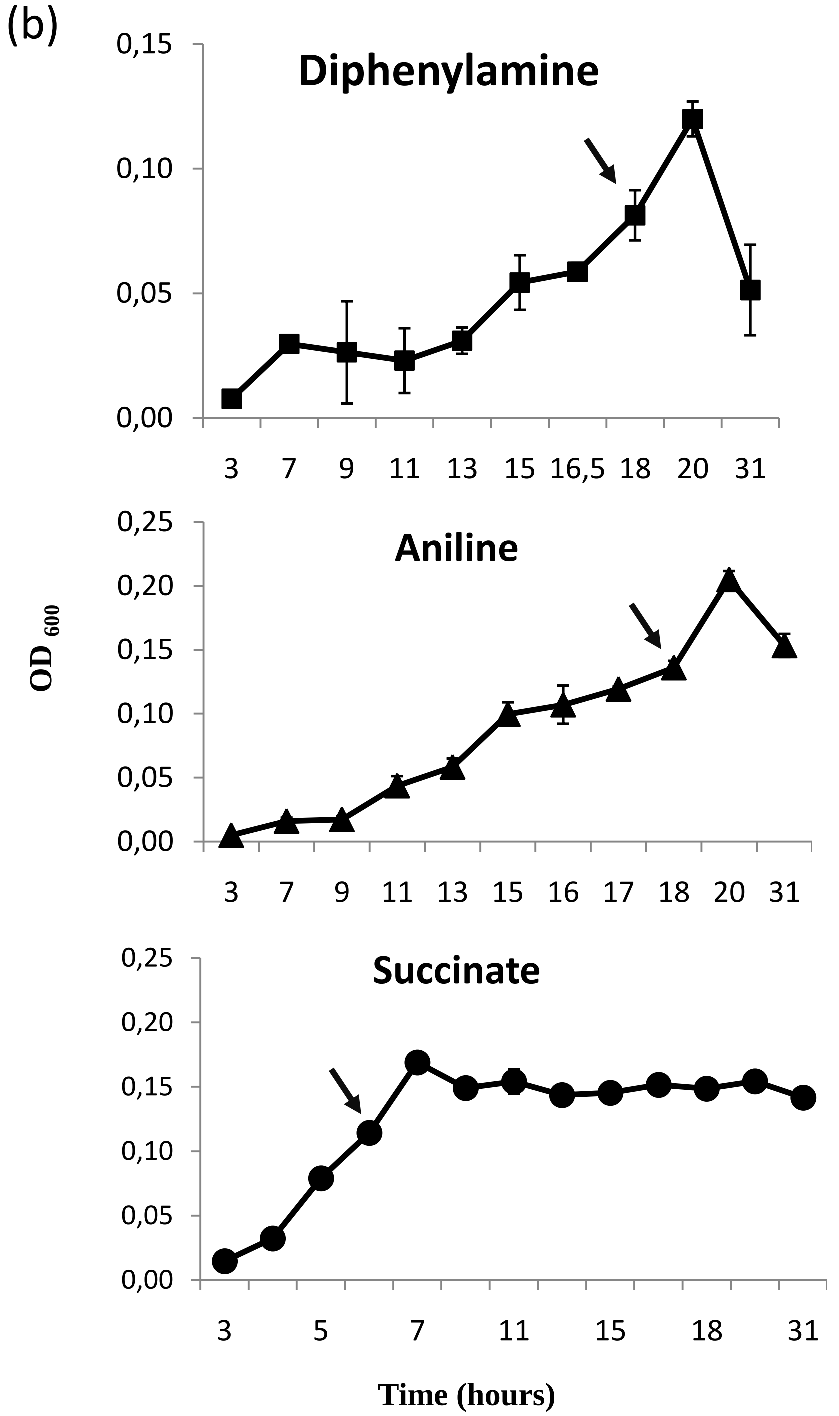

Supplementary Figure 3: (a) The degradation of diphenylamine (DPA) and aniline in MSMN inoculated (closed symbols, solid lines) or not (open symbols, dashed lines) with *P. putida* DPA1 and (b) the growth of *P. putida* in MSMN amended with DPA, aniline or succinate as determined by OD_{600}. Arrows indicate the time point in the late mid-log phase of bacterial growth when bacterial pellet for protein extraction and subsequent proteomic analysis was collected. Each value is the mean of three replicates ± the standard deviation.

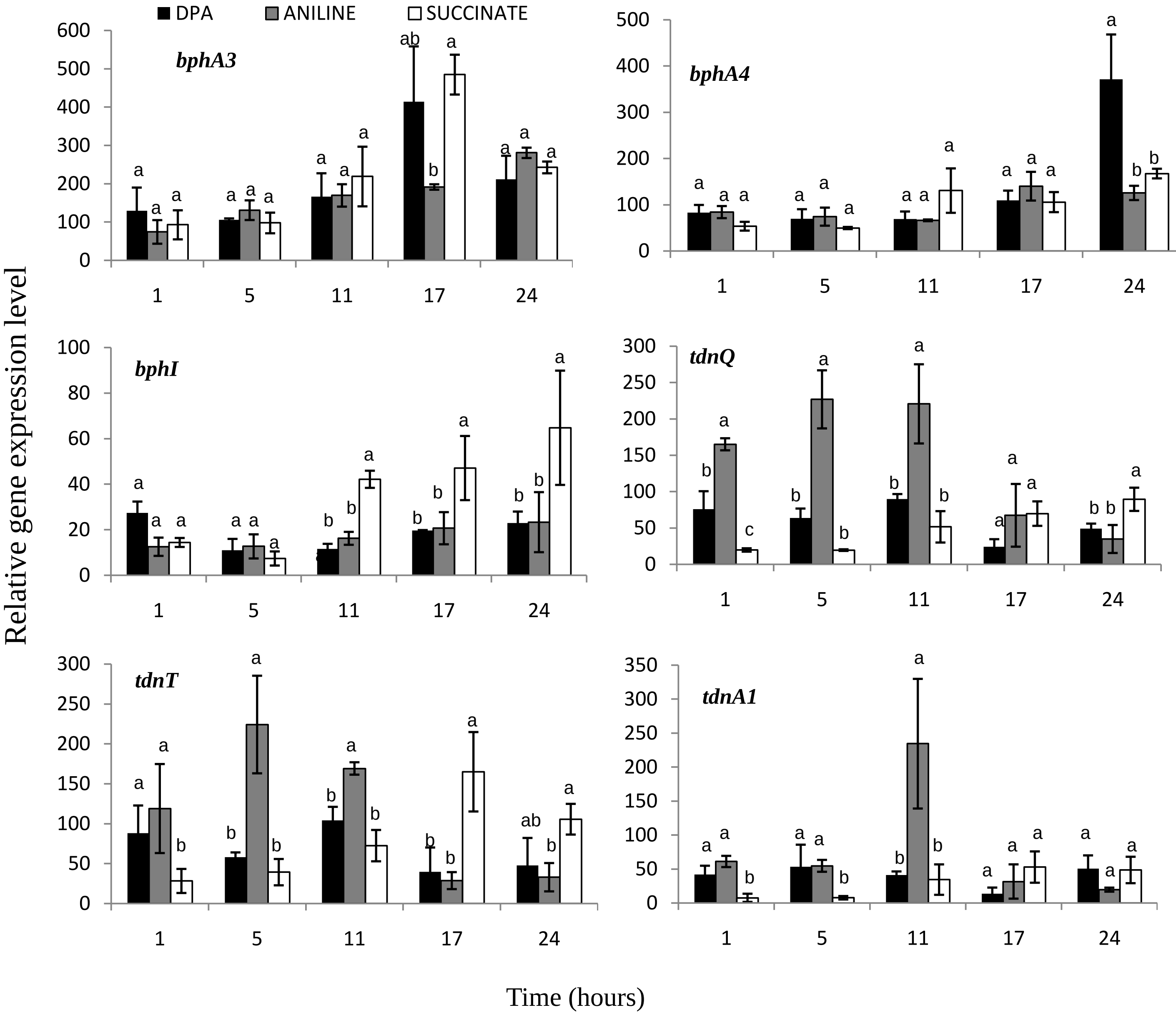

Supplementary Figure 4. The transcriptional pattern of the genes *bphA3A4*, *bphI* (located in the *bph* operon 1) and *tdnQTA1* (located in the *tdn* operon 2) in *P. putida* strain DPA1 cells growing in MSMN amended with diphenylamine (DPA), aniline or succinate. Each value is the mean of three replicates $\pm$ the standard deviation. Within each time point bars designated by the same letter are not significantly different at the 5% level.

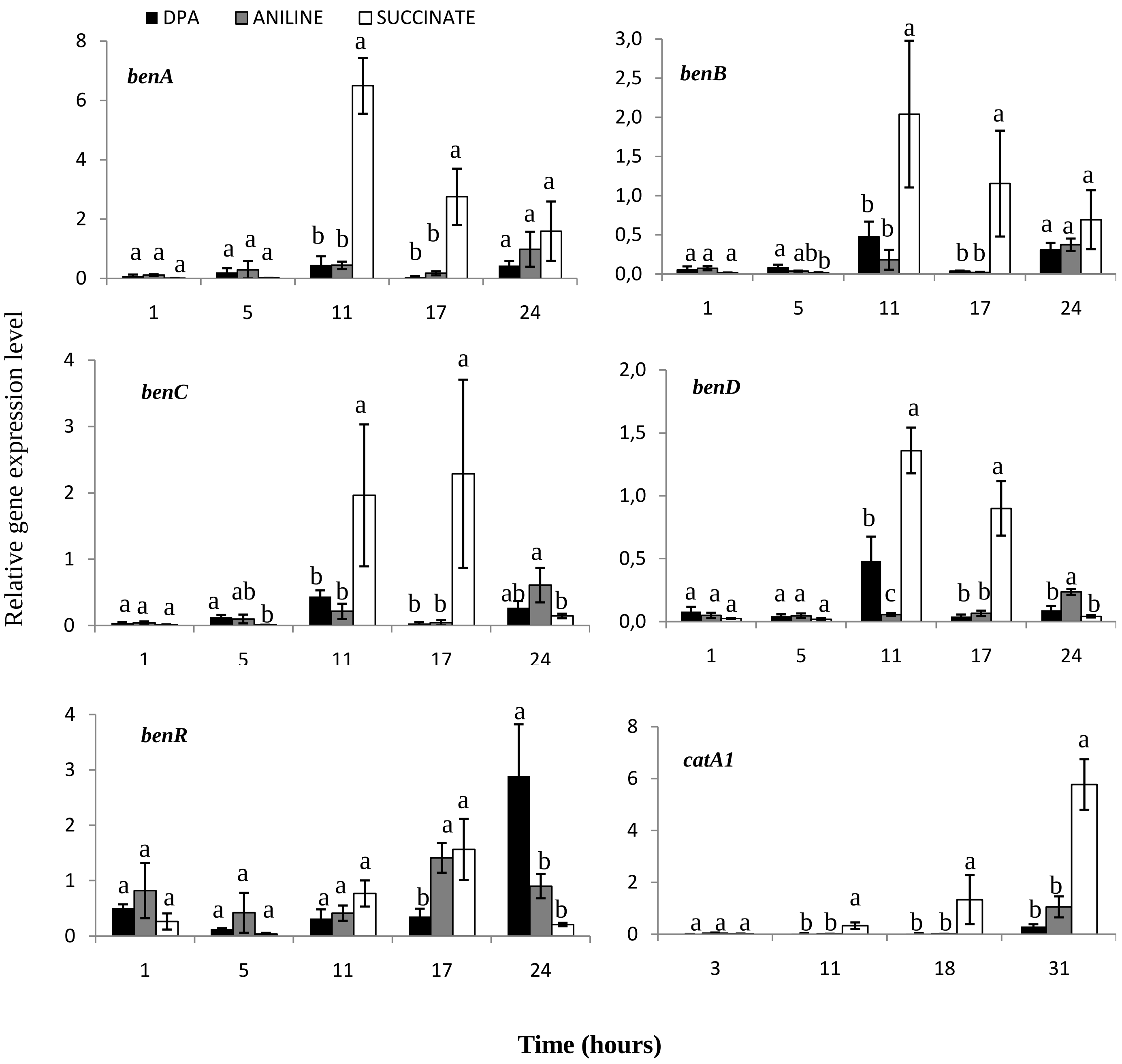

Supplementary Figure 5. The transcriptional patterns of genes *benABCDR* and *catA1* genes from operon 5 in the *P. putida* strain DPA1 growing in MSMN amended with diphenylamine (DPA), aniline or succinate. Each value is the mean of three replicates + the standard deviation. Within each time point bars designated by the same letter are not significantly different at the 5% level.

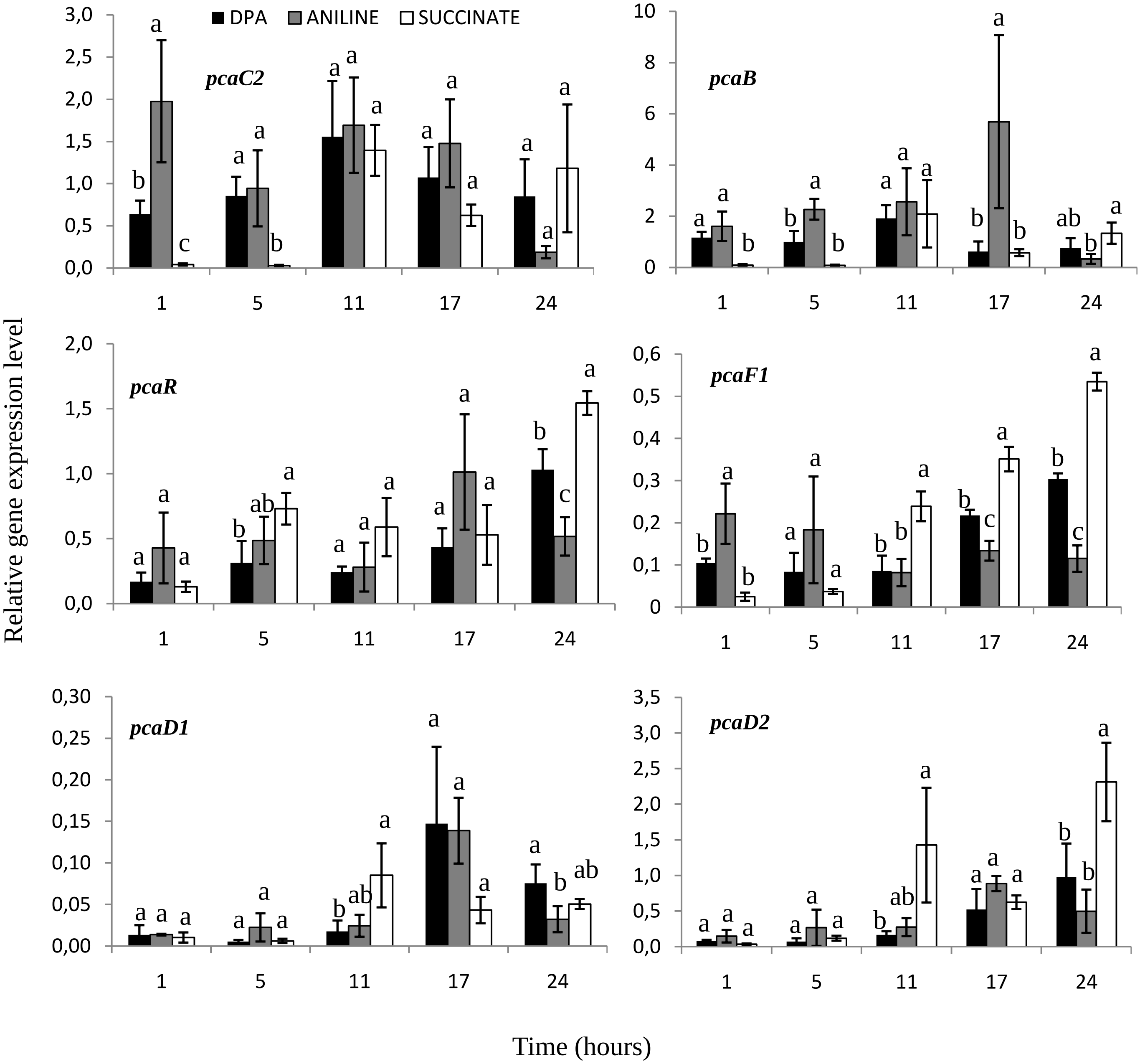

Supplementary Figure 6. The transcriptional patterns of the genes *pcaC2BR* genes of operon 5 and *pcaF1*, *pcaD1* and *pcaD2* (localized in Scaffold 1 but not organized in an operon) in cells of *P. putida* strain DPA1 growing in MSMN amended diphenylamine (DPA), aniline or succinate. Each value is the mean of three replicates ± the standard deviation. Within each time point bars designated by the same letter are not significantly different at the 5% level.

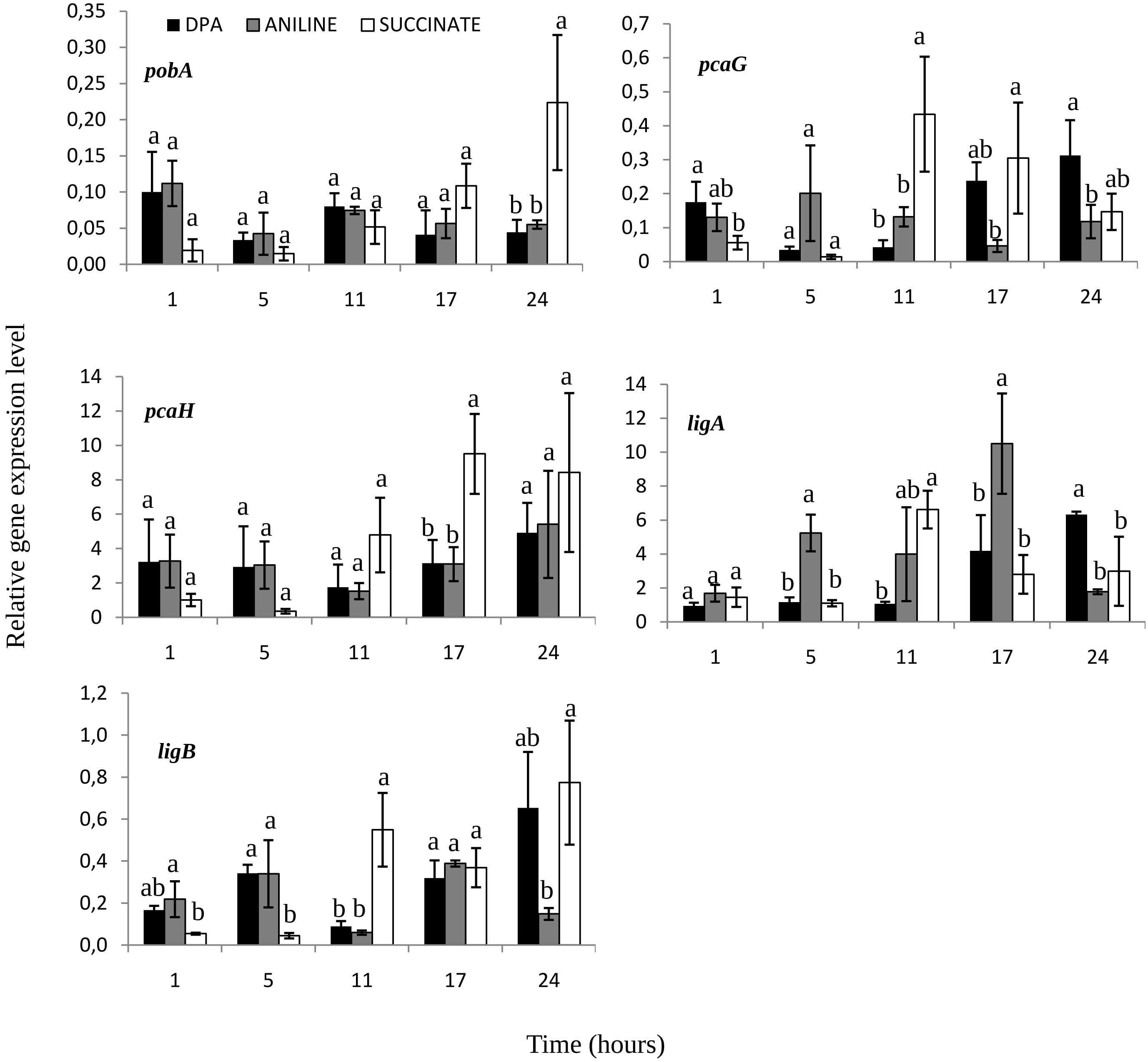

Supplementary Figure 7. The transcriptional patterns of genes *pobA*, *pcaGH* and *ligAB*, whose annotation indicate a putative catabolic role in transformation of aromatic compounds, in cells of the *P. putida* strain DPA1 growing in MSMN amended with diphenylamine (DPA), aniline or succinate. Each value is the mean of three replicates ± the standard deviation. Within each time point bars designated by the same letter are not significantly different at the 5% level.

1.2. **Supplementary Tables**

Supplementary Table 1. The primers used for the transcriptional analysis of the putative catabolic genes. The size of the obtained amplicons is also given. Letters "f" and "r" indicate respectively the forward and the reverse primer.

Target Gene	Gene Code	Primer sequence (5'-3')	Amplicon size(bp)
gyrB	CBL13_4912	f AGCCTGCGTGAAGACAAAGAG	239
		r TCGTTCATCAGCCAATCCAG	
bphI	CBL13_03816	f CCGATGTGGATGAGGTGATT	154
		r GTGAACTGCAAAATGGCGTA	
bphA1	CBL13_03823	f ATCTTTGCGCGCTCCTGGCT	153
		r ACGGTGGCGGCATTGGTTC	
bphA2	CBL13_03824	f GAGCTGAACTGGGCCGAAGACC	238
		r TGCTCTGGTCGATCAGGATGGTG	
bphA3	CBL13_03825	f ATGACGGGCAAAAGGTGGCGAT	173
		r CGACTTGACCTTGCCTGTGCGTA	
bphA4	CBL13_03827	f AAGCCCCTGTCGTCGTCCT	184
		r CAGTCGAGGCGGATTTTCTC	
bphR	CBL13_03828	f ACGCGTAGCACACAACTGAC	152
		r GTAATGCCGTCGGATAAAGC	
tdnQ	CBL13_05652	f GATACCATTCAAGTTCAACCCGTG	197
		r GCTGCTTCTAAACCTTCCATTACATC	
tdnT	CBL13_05653	f TACCAGTGAAGAGCGAGTGAGGC	198
		r AGATGAACCCGTAGAAAGACTTGC	
tdnA1	CBL13_05654	f CAGCAAGAGATTTAGAGCGAGC	222
		r CGTCATCCACCTCACCCATA	
tdnA2	CBL13_05655	f CGCTGTTAACCTCGCTTATTATCA	188
		r TCCAGTAACAGCCATCCTCCA	
tdnB	CBL13_05656	f GTTATTTGCAGGTGGCAGTGGTA	205
		r TGGCGTTCCCTGTTCATCC	
tdnR	CBL13_05657	f ACCCCAGGTACTGAAATCATACTC	227
		r CCTTCCCGCTCCAAATCAAC	
benA	CBL13_01122	f CTACGAAGGCAACTGGAAGC	186
		r CCGTGGTCGAAGGAATAGAA	
benB	CBL13_01123	f AAGACCCACAAAGCGAAATC	240
		r AAAAGCTGGTGCCGAAGTAA	

benC	CBL13_01124	f	GAATTTCGAAGACGGGGTTA	199
		r	GATTTCGTCTTCGCTCAAGG	
benD	CBL13_01125	f	GTCAACCGTGTGCCCTATG	185
		r	TGGTACCAGTGCTTTTCCTG	
benD	CBL13_01135	f	ATCAAGGGCGATGAAGTCTG	217
		r	TCACGAATTTGCTCAGTGCT	
catA	CBL13_01127	f	CCACGCCTCAAACGCATC	180
		r	CAGCAGGTCGAGGAAGTGTTC	
catA	CBL13_01888	f	GGCGACTTGGCACCCTGTA	229
		r	CGCACGTGCACTTTTTCATCTC	
catC	CBL13_01889	f	AAGGCCGATGAAAAGGAACT	218
		r	CGATCGTCGCTGTGAATAGA	
catB	CBL13_01890	f	GATCTCCTCGGTCAGCAGCAG	208
		r	CTTCGCCCTGAAAATCGCCA	
catR	CBL13_01891	f	AGTGGGCCAACGAACTACAG	185
		r	CTTCACGATCGGGCTTACAT	
pcaC	CBL13_03869	f	CGTCATACCCGTAGCCTGAT	203
		r	AGTTCATCCCACACCGACTC	
pcaD	CBL13_03870	f	TCAGCGCGGGCACCTGGAT	188
		r	CGACCTGCGCGATGCCTCTATC	
pcaB	CBL13_03871	f	AACAGCGTCGACAGCAAGCC	229
		r	GCGACCGCCTGGTGGAGTT	
pcaF	CBL13_03873	f	GTCGGCACGCGAAACCTGAT	225
		r	AGCGGCGAGATGGAACTGG	
pcaR	CBL13_03875	f	ACCAGCCTGCGTGAGTATCT	208
		r	TTACGTTCAAGGCAGCCAAC	
pcaD	CBL13_01483	f	GAGTGGCCGGTATTCAGTGT	209
		r	ACATTCGCCGAAATCAGTG	
pcaD	CBL13_01858	f	CTTATTCGCTGTTCGCCACT	223
		r	TCCAGCAGCATCTTGTTGAC	
pcaF	CBL13_01220	f	TGACATCCAGGTCAGCCAGTG	243
		r	GGACGCCGTTTCGTGAAGG	
pcaI	CBL13_02043	f	CAACCTGGCCGAGCGTAT	165
		r	GCCTTGATCAGTGCGAAGTC	
pcaJ	CBL13_02044	f	GCCAGGTGTTCGTGATGATG	194
		r	CTTCTGCAGCTCGTCAAAATC	

pobA	CBL13_00737	f	CGCATTCTGGTCAAGGTGTA	233
		r	CCCACATAGTTCTCGGCAAT	
pcaG	CBL13_04043	f	GCACATCAACATCAGCCTGT	222
		r	AAGAAAACGGTCTCCCCTTC	
pcaH	CBL13_04044	f	AGCCCTTACGCCTGACTACA	164
		r	GCAGCCCACCATTATTGAAG	
ligA	CBL13_00562	f	ATCCTGGTTGACATCAGTGC	236
		r	GGCCTCAATCGACAGAACAG	
ligB	CBL13_00004	f	GTGCCACTGTCGCTGATGTA	240
		r	GTCTACCACCCAATCCCTGA	

Supplementary Table 4. A list of proteins, listed according to their general function, identified via proteomic analysis to be significantly (p<0.05) up-regulated in *P. putida* DPA1 cells grown on aniline (ANI) and DPA compared to cells grown on succinate. Asterisks next to spots intensity ratio values indicate the level of statistical significance (*, **, *** for p<0.05, p<0.01 and p<0.001 respectively) in differences in the spot intensity between DPA, ANI and succinate.

Spot No.	Proteins	Spots intensity ratio	
		ANI/Succ	DPA /Succ
	Stress-related proteins		
1014	Alkyl hydroperoxide reductase subunit C	2.15*	5.26***
2005	Putative peroxiredoxin	1.4	34.62**
2007	Thiol peroxidase	2.65*	2.48**
3004	DNA protection during starvation protein (Dps)	4.25	16.71***
8003	Small heat shock protein IbpB	1.79	6.12**
	Transporters and membrane-related proteins		
517	Porin D precursor	4.7***	6.2***
5508	Porin D precursor	1.16	3.58***
3312	Spermidine/putrescine-binding periplasmic protein precursor	5.33***	8.91***
8411	Putrescine-binding periplasmic protein precursor	23.12**	76.22***
8309	Putrescine-binding periplasmic protein precursor	13.71*	24.78***
8004	Protein YceI	1.19	3.27***
605	Sulfate-binding protein precursor	2.1***	2.01***
4209	General L-amino acid-binding periplasmic protein AapJ precursor	5.24***	3.97***
4212	General L-amino acid-binding periplasmic protein AapJ precursor	3.22	10.05***
5218	General L-amino acid-binding periplasmic protein AapJ precursor	5.19*	19.74***
4408	Leucine-, isoleucine-, valine-, threonine-, and alanine-binding periplasmic protein	11.51***	12.05***
4409	Leucine-, isoleucine-, valine-, threonine-, and alanine-binding periplasmic protein	4.41***	13.32***
5412	Leucine-, isoleucine-, valine-, threonine-, and alanine-binding periplasmic protein	4.73***	5.72***
5415	Leucine-, isoleucine-, valine-, threonine-, and alanine-binding periplasmic protein	5.32***	4.19***
5409	Leucine-, isoleucine-, valine-, threonine-, and alanine-binding periplasmic protein	2.39***	3.11***
2101	D-ribose-binding periplasmic protein precursor	4.11***	6.54***
5216	Tripartite tricarboxylate transporter family receptor	6.24*	23.1***

8106	Membrane lipoprotein TpN32 precursor	2.42**	2.46***
8107	Cystine-binding periplasmic protein precursor	14.43**	14.9***
6114	Lysine-arginine-ornithine-binding periplasmic protein precursor	7.52	14.19**
8118	Lysine-arginine-ornithine-binding periplasmic protein precursor	7.66***	7.62***
	Energy production and biosynthesis proteins		
4411	Isocitrate dehydrogenase [NADP] (EP)[a]	1.13	2.43**
4412	Isocitrate dehydrogenase [NADP] (EP)	2.7	7.53***
5416	Isocitrate dehydrogenase [NADP] (EP)	4.62***	6.63***
4709	Phosphoglucomutase (EP)	1.25	2.65***
5106	Succinyl-CoA ligase [ADP-forming] subunit alpha (EP)	1.48	4.03***
8418	Succinyl-CoA ligase [ADP-forming] subunit beta (EP)	1.29	2.38*
5611	Aldehydedehydrogenase PuuC (EP)	2.96*	2.44**
3714	Phosphoenolpyruvatecarboxykinase [ATP] (EP)	1.51**	2.48***
3608	ATP synthase subunit alpha (EP)	1.12	7.26***
5610	Aldehyde dehydrogenase B (EP)	1.76*	2.28***
5612	Aldehyde dehydrogenase B (EP)	1.29	6.15***
6609	Aldehyde dehydrogenase B (EP)	5.26**	4.59**
6610	Aldehyde dehydrogenase B (EP)	3.32*	5.53**
6707	Aldehyde dehydrogenase B (EP)	2.1*	1.98
6207	NH(3)-dependent NAD(+) synthetase (EP)	1.94**	2.54***
8016	Azurin precursor (EP)	4.03***	4.58***
4510	Argininosuccinate synthase (AB)	1.88**	2.36***
6306	Aromatic-amino-acid aminotransferase (AB)	0.89	2.34*
9304	Gamma-glutamyltranspeptidase precursor (AB)	17.79***	41.38***
4004	3-isopropylmalate dehydratase small subunit 1 (AB)	2.18*	6.54***
2009	Aspartokinase (AB)	1.45	3.2**
3513	Aspartokinase (AB)	1.47	5.24*
2308	Elongation factor Tu (PB)	5.17*	1.88*
2712	Proline-tRNA ligase (PB)	0.92	2.72***
5411	Phosphoribosylglycinamide formyltransferase 2 (NB)	1.27	2.05**
2109	Orotatephosphoribosyltransferase (NB)	0.74	2.48*
4310	3-oxoacyl-[acyl-carrier-protein] synthase (FB)	3.26**	4.00***
5507	1-deoxy-D-xylulose 5-phosphate reductoisomerase	1.01	4.25*
4112	glutamine amidotransferase	8.55*	1.82

[a] EP: Energy Production; AB: Amino acids Biosynthesis; PB: Protein Biosynthesis; DB: DNA biosynthesis; FB: Fattyacid Biosynthesis; NB: Nucleotide Biosynthesis.
